# Inhibition of glypican-1 expression induces an activated fibroblast phenotype in a human bone marrow-derived stromal cell-line

**DOI:** 10.1038/s41598-021-88519-7

**Published:** 2021-04-29

**Authors:** Sukhneeraj P. Kaur, Arti Verma, Hee. K. Lee, Lillie M. Barnett, Payaningal R. Somanath, Brian S. Cummings

**Affiliations:** 1 Department of Pharmaceutical and Biomedical Sciences, College of Pharmacy, University of Georgia, 450 College of Pharmacy South, Athens, GA 30607 USA; 2 Clinical and Experimental Therapeutics, University of Georgia and Charlie Norwood VA Medical Center, Augusta, GA USA; 3 Medical College of Georgia, Augusta University, Augusta, GA USA; 4 Interdisciplinary Toxicology Program, University of Georgia, Athens, GA USA; 5 Department of Medicine and Vascular Biology Center, Augusta University, Augusta, GA USA

**Keywords:** Cancer, Urological cancer, Prostate cancer

## Abstract

Cancer-associated fibroblasts (CAFs) are the most abundant stromal cell type in the tumor microenvironment. CAFs orchestrate tumor-stromal interactions, and contribute to cancer cell growth, metastasis, extracellular matrix (ECM) remodeling, angiogenesis, immunomodulation, and chemoresistance. However, CAFs have not been successfully targeted for the treatment of cancer. The current study elucidates the significance of glypican-1 (GPC-1), a heparan sulfate proteoglycan, in regulating the activation of human bone marrow-derived stromal cells (BSCs) of fibroblast lineage (HS-5). GPC-1 inhibition changed HS-5 cellular and nuclear morphology, and increased cell migration and contractility. GPC-1 inhibition also increased pro-inflammatory signaling and CAF marker expression. GPC-1 induced an activated fibroblast phenotype when HS-5 cells were exposed to prostate cancer cell conditioned media (CCM). Further, treatment of human bone-derived prostate cancer cells (PC-3) with CCM from HS-5 cells exhibiting GPC-1 loss increased prostate cancer cell aggressiveness. Finally, GPC-1 was expressed in mouse tibia bone cells and present during bone loss induced by mouse prostate cancer cells in a murine prostate cancer bone model. These data demonstrate that GPC-1 partially regulates the intrinsic and extrinsic phenotype of human BSCs and transformation into activated fibroblasts, identify novel functions of GPC-1, and suggest that GPC-1 expression in BSCs exerts inhibitory paracrine effects on the prostate cancer cells. This supports the hypothesis that GPC-1 may be a novel pharmacological target for developing anti-CAF therapeutics to control cancer.

## Introduction

Solid cancers are composed of two distinct, yet interdependent, compartments: the tumor cells and the surrounding stroma. Tumor stroma is a complex repertoire of immune cells, endothelial cells, fibroblasts, pericytes, mesenchymal stem cells (MSCs), and extracellular matrix (ECM)^1^. Bidirectional interaction between cancer cells and the associated stroma is essential for tumor growth and progression. Cancer-associated fibroblasts (CAFs) are important components of tumor stroma that orchestrate transformations in the tumor milieu, influencing cancer pathology by affecting its initiation, progression and chemoresistance^2–4^. CAFs isolated from prostate cancer tumors can enhance tumorigenicity of both initiated and non-tumorigenic prostate epithelial cells^5,6^. Several cell precursors contribute to the development of CAFs, such as MSCs, fibroblasts, bone marrow-derived stromal cells (BSCs), epithelial cells and endothelial cells^3,7^. As such, CAFs have been proposed as a cellular state rather than a cell-type^8^. These various CAF progenitors contribute to CAF heterogeneity, giving rise to several distinct subpopulations in the tumor microenvironment (TME) of solid cancers. These subtypes are functionally distinct, exerting tumor-promoting and tumor-inhibiting influences in the TME^9–11^. The incontrovertible role of CAFs in cancer progression highlights the need to develop CAF-targeted therapies for improving the outcomes in cancer patients.

Glypicans are cell surface heparan sulfate proteoglycans (HSPGs) that are bound to the exocytoplasmic surface of cell membranes via a glycosylphosphatidylinositol (GPI) linkage. The human genome consists of six different isoforms of glypicans, referred to as *GPC-1* to *GPC-6*, which encode for six different proteoglycans^12,13^. Glypicans interact with a wide range of soluble and insoluble ligands, enabling them to impact essential aspects of cellular fate, including cell growth, migration and differentiation^12–15^. They regulate several cell signaling pathways involving morphogens^16,17^, growth factors^18–21^, and ECM proteins^22–25^.

Glypicans’ expression is differentially altered in several cancers, and exhibit a context-dependent inhibition and promotion of cancer growth. Therefore, it is important to understand their role in contributing to the pleiotropism and plasticity of the TME. A few studies have determined the role of glypicans in regulating various players of the TME; however, their role in the activation of CAFs has not been investigated^26^. Addressing this gap-in-knowledge and elucidating the molecular mechanisms involved in the activation of CAFs is crucial to understanding the complexity that surrounds the tumor-stroma relationship. Identification of these mechanisms will also aid in identifying therapeutic targets for prevention of cancer cell growth and metastasis.

GPC-1 has been proposed to be a biomarker in prostate cancer^27^, and our recent study shows that GPC-1 displays opposing roles in the progression of prostate cancer by acting as a tumor promoter in 2D models of prostate cancer cell growth, but inhibiting tumor growth in vivo, possibly by interacting with different components of the TME^28^*.* Furthermore, this same study showed that treatment of fibroblasts and human MSCs with conditioned media isolated from prostate cancer cells in which GPC-1 was inhibited, increased the expression of genes, proteins and enzymes known to facilitate tumor growth^28^. This study suggested that the lack of GPC-1 in prostate cancer cells induces a more reactive tumor stroma in vivo.

While our recent study suggested that GPC-1 acts as a tumor suppressor, it is not known if GPC-1 plays a direct role in modulating the activation of stromal cell components, such as fibroblasts, in prostate cancer. It is to be noted that GPC-1 mRNA expression is reduced in primary tumors in patients with prostate cancer as compared to normal tissue^28^. Similar results were obtained using the Gene Expression Profiling Interactive Analysis (GEPIA) database, which showed that GPC-1 is under-expressed in prostate adenocarcinoma^29^. Kaplan–Meier curve analysis showed a positive correlation between the GPC-1 expression and the survival rate in prostate cancer patients. Therefore, patients with relatively low GPC-1 expression had a lower survival rate in comparison to patients with higher levels of GPC-1^28,30^. Furthermore, the protein expression of GPC-1 is low to undetectable in prostate cancer tissue samples^30^.

Based on the above studies, we tested the hypothesis that GPC-1 modulates activated fibroblast behavior. We tested this hypothesis by creating a GPC-1 knockdown human BSC cell-line and assessed the impact of loss of GPC-1 expression on the induction of activated fibroblast-like behavior of BSCs. We chose a human bone-derived stromal cell-line of fibroblast lineage, HS-5, as bone is one of the most prominent sites for organ metastasis in prostate cancer^31^. We further studied how these activated fibroblasts affect the proliferation of prostate cancer cells after they have settled in the bone microenvironment. We hypothesized that the loss of GPC-1 in HS-5 cells would support a more aggressive phenotype in the bone-metastasized prostate cancer cells.

## Materials and methods

### Cell culture

HS-5, HS-27, PC-3, DU-145 and RM1 cell-lines were obtained from American Type Culture Collection (ATCC, Manassas, VA). HS-5 (human bone-derived stromal cells of fibroblast lineage) and HS-27 (human foreskin-derived fibroblasts) cells were cultured in complete growth medium containing DMEM (ATCC), 10% FBS (Seradigm, Radnor, PA) and 1% Pen/Strep (ATCC, Manassas, VA). PC-3 (androgen insensitive, human bone-derived metastatic prostate cancer cells), DU-145 (androgen insensitive, human brain-derived metastatic prostate cancer cells) and RM1 (murine metastatic prostate cancer cells) cells were cultured in complete growth medium consisting of RPMI-1640 (Corning Inc., Corning, NY) supplemented with 10% FBS and 1% Pen/Strep. All cultures were maintained in 5% CO_2_ and 95% humidity at 37 °C.

### Lentiviral transduction

Stable knockdown of GPC-1 was developed in HS-5 cells. Control and GPC-1 shRNA lentiviral particles were purchased from Sigma (SHC002V, TRCN0000122909, TRCN0000122911). Sequences of shRNA particles are provided at the manufacturer’s website (https://www.sigmaaldrich.com/catalog/genes/GPC1). HS-5 cells (1.02 × 10^4^ per well) were seeded in a 96-well plate. Complete growth media (DMEM) was used for all transductions. All treatments were done in duplicate. After overnight incubation, each well was treated with 50 μL complete growth media containing 16 μg/mL polybrene (Cell Signaling Technology, Danvers, MA). Volumes of lentiviral particles with a multiplicity of infection of 5 was calculated for each shRNA construct per well and mixed in 50 μL media and added to appropriate wells. After 24 h, media containing lentiviral particles were aspirated and replaced with 200 μL of fresh media. After another 24 h, cells were selected using 0.5 μg/mL puromycin, followed by the expansion of each clone. qRT-PCR analysis and western blot analysis was performed on control and GPC-1 shRNA cells to confirm the knockdown. Two different clones of GPC-1 knockdown were successfully developed in HS-5 cells using shRNA TRCN0000122909 and TRCN0000122911, referred to as GPC-1 shRNA HS-5 clone A and clone B, respectively.

### Collection of cell conditioned media (CCM)

CCM were collected from PC-3 and DU-145 cells by growing these cells in 10-cm dishes (1 × 10^6^ cells/dish) in complete growth media (RPMI). After 24 h or when the cells reached about 90% confluence, they were washed twice with 1X PBS and incubated in 8 mL serum-free DMEM. DMEM was used to collect CCM from PC-3 and DU-145, since HS-5 cells demonstrated sensitivity to RPMI media (Supplementary Fig. 1). Hence, all experiments requiring incubation of HS-5 cells with prostate cancer CCM were performed using DMEM. CCM was collected after 24 h, centrifuged at 1000×*g* for 10 min, and passed through a 0.22 μm filter to eliminate cell floaters and debris. Serum-free CCM was used for all experiments, unless otherwise specified. A similar approach was used for collecting BSC CCM, except that control and GPC-1 knockdown HS-5 cells (1 × 10^6^ cells/dish) were used in place of prostate cancer cells.

### Total RNA isolation, cDNA synthesis and quantitative real-time polymerase chain reaction (qRT-PCR)

Control and GPC-1 knockdown HS-5 cells (1 × 10^5^ cells/well in 1 mL complete media) were seeded in 24-well plates. After 24 h, complete media was replaced with 1 mL serum-free media after washing twice with 1X PBS. Total RNA was isolated after an overnight incubation. A similar protocol was followed for PC-3 cells treated with HS-5 CCM. Briefly, PC-3 cells (1 × 10^5^ cells/well in 1 mL complete media) were plated in 24-well plates. After overnight incubation, PC-3 cells were washed twice with 1X PBS and were incubated in 1 mL CCM isolated from control and GPC-1 knockdown HS-5 cells, followed by RNA isolation after 24 h. Total RNA was isolated from cells using TRIzol™ Reagent (Invitrogen, Carlsbad, CA) according to the manufacturer’s protocol. The quantity and purity of RNA was assessed using a NanoDrop 2000/2000c spectrophotometer to determine A260/A280 and A260/A230 ratios (Thermo Scientific, Waltham, MA). cDNA (total volume: 20 μL) was synthesized from 2 μg of RNA using the high-capacity cDNA reverse transcription kit (Applied Biosystems, Foster City, CA), following the manufacturer’s directions. cDNA was diluted 10 times with diethyl pyrocarbonate (DEPC)-treated water (Fisher Scientific, Hampton, NH) before proceeding for qRT-PCR.

The expression of genes listed in Table 1 was analyzed using qRT-PCR. The primers (Table 1) were purchased from Integrated DNA Technologies (Coralville, IA), and have been validated and used in previous studies from our laboratories^28^. All experiments were performed using the SYBR Select Master Mix kit (Applied Biosystems, Foster City, CA) on a QuantStudio 5 RT-PCR System (Applied Biosystems, Foster City, CA). Fold difference was calculated using the 2^−ΔΔCT^ method. GAPDH was used as an internal control for normalization.

**Table 1:** List of genes and primers used in the study.

Genes	Primers
Glypican-1	F: 5′-CTTAGTGCTGCTTTGCTTTTCAT-3′ R: 5′-AGGGTTATTATGGGGTGGACTT-3′
Glypican-2	F: 5′-TTAGGAGGGAGTGTGGTTTCC-3′ R: 5′-AAAACTCAACAGAACCCAGGC-3′
Glypican-3	F: 5′-CATGTCTATGCCCAAAGGTAGAG-3′ R: 5′-ATCATCCACATCCAGATCATAGG-3′
Glypican-4	F: 5′-AAGCTGTCTTTGCTTCACGTTAC-3′ R: 5′-TAGCATTTCTTCCAGGTTCACAT-3′
Glypican-5	F: 5′-GGTGTGACTGACAGTTCCCTG-3′ R: 5′-TGCAGATAGTCTGTGGTGTTGAT-3′
Glypican-6	F: 5′-CCCAAGACAGCTACATTTTCAAC-3′ R: 5′-ATACCTCCAAGACAACAGTGCAT-3′
GAPDH	F: 5′- AGCCACATCGCTCAGACAC -3′ R: 5′-TGGAAGATGGTGATGGGATT-3′
IL-6	F: 5′-ACTCACCTCTTCAGAACGAATTG-3′ R: 5′-CCATCTTTGGAAGGTTCAGGTTG-3′
IL-8	F: 5′-GGCACAAACTTTCAGAGACAG-3′ R: 5′-ACACAGAGCTGCAGAAATCAGG-3′
N-Cadherin	F: 5′-CCATCAAGCCTGTGGGAATC-3′ R: 5′-CTGTGGGGTCATTGTCAGCC-3′
E-Cadherin	F: 5′-CTCGTAACGACGTTGCACC-3′ R: 5′-CTGTGGGGTCAGTATCAGCC-3′
Zeb-1	F: 5′-TTCAAACCCATAGTGGTTGCT-3′ R: 5′- TGGGAGATACCAAACCAACTG-3′
Zeb-2	F: 5′-GCACAAGACTACATGTCAGGCC-3′ R: 5′-CACACTGATAGGGCTTCTCGC-3′
CXCR4	F: 5′-ACGTCAGTGAGGCAGATG-3′ R: 5′-GATGACTGTGGTCTTGAG-3′
MMP-9	F: 5′-CAACATCACCTATTGGATCC-3′ R: 5′-CGGGTGTAGAGTCTCTCGCT-3′
Vimentin	F: 5′-CTGGATTCACTCCCTCTGGTTG-3′ R: 5′- GGTCATCGTGATGCTGAGAAG-3′

### Immunoblot analysis

Control and GPC-1 knockdown HS-5 cells (1 × 10^5^ cells/well in 1 mL complete media) were seeded in 24-well plates. After 24 h, complete media was replaced with 1 mL serum-free media after washing twice with 1X PBS. Total protein was isolated after overnight incubation. For the isolation of protein from wild-type HS-5 cells exposed to prostate cancer CCM, wild-type HS-5 cells (1 × 10^5^ cells/well in 1 mL complete media) were plated in 24-well plates. After overnight incubations, cells were washed twice with 1X PBS and exposed to 1 mL of DU-145 CCM or PC-3 CCM for 48 h, followed by protein isolation and immunoblot analysis as per standard protocols. CCM was replaced with fresh CCM after 24 h. All protein samples were collected in RIPA buffer in the presence of a protease/phosphatase inhibitor cocktail (100X, Cell Signaling Technology, Danvers, MA).

Primary antibodies used included GPC-1 (62 kDa; 1:1000, GeneTex, cat. no. GTX104557), phosphorylated-extracellular signal regulated kinase (ERK) (44/42 kDa; 1:1000, Cell Signaling Technology, cat. no. 9101), total-ERK (44/42 kDa; 1:1000, Cell Signaling Technology, cat. no. 9102), vimentin (57 kDa; 1:1000, Santa Cruz, sc6260), alpha-smooth muscle actin (42 kDa; 1:200, Abcam, ab5694) and GAPDH (37 kDa; 1:4000, Santa Cruz, sc32233). A suitable horseradish-peroxidase (HRP)-conjugated anti-mouse and anti-rabbit antibody (Promega Corporation, Madison, WI) were used as secondary antibodies at the dilution of 1:2500. Protein bands were visualized using SuperSignal™ West Pico chemiluminescent substrate (Thermo Scientific, Waltham, MA) utilizing an Alpha Innotech FluorChem HD2 system (ProteinSimple, San Jose, CA). Data was analyzed by densitometric analysis of protein bands using FluoChem HD2 software. GAPDH was used as the endogenous control.

### Crystal violet staining

Crystal violet staining was used to determine the rate of cell growth of HS-5 cells after GPC-1 knockdown. Cells were initially seeded in 6-well plates (5 × 10^4^ cells/well) for 48 h, after which they were fixed in 4% (v/v) formaldehyde (Sigma, St. Louis, MO), followed by incubation with 1% (w/v) crystal violet. Cells were washed after 15 min and the plates were air dried overnight. Crystal violet staining was visualized under phase contrast microscopy using a Nikon Eclipse T*i* inverted microscope (Nikon, Tokyo, Japan). HS-5 cell morphology after GPC-1 knockdown was observed after seeding of the cells in 6-well plates (2 × 10^5^ cells/well) for 24 h, followed by crystal violet staining. Crystal violet staining was also performed on GPC-1 knockdown HS-5 cells after exposure to prostate cancer CCM. Cells were exposed to prostate cancer CCM for 24 h, and stained using the protocol above. In some cases, such as that for HS-5 cells after exposure to prostate cancer CCM, and PC-3 cells after exposure to HS-5 CCM, cells were directly observed under the phase contrast microscope without the stain after 24 h.

### DAPI staining

Nuclear morphology was assessed using 4′,6-diamidino-2-phenylindole (DAPI). Control and GPC-1 knockdown HS-5 cells (5 × 10^4^ cells in 1 mL complete media) were seeded in 24-well plates. After an overnight incubation, cells were fixed in 4% (v/v) formaldehyde, washed with 1X PBS, followed by incubation in PBS containing DAPI (stock: 1.5 μg/mL, Vector Laboratories, Burlingame, CA) for 10 min in the absence of light, followed by two washes with 1X PBS. Cells were imaged using the Carl Zeiss inverted fluorescent digital microscope (Carl Zeiss MicroImaging Inc., Oberkochen, Germany). The nuclear size was determined by quantifying nuclear area using ImageJ software.

### MTT assay

Cell proliferation was assessed based on the reduction of 3-(4,5-dimethylthiazolyl-2)-2,5-diphenyltetrazolium bromide (MTT). HS-5 cells were plated (3 × 10^4^ cells/well in 400 μL complete media) in 48-well plates, and the difference in cell proliferation between HS-5 control and GPC-1 knockdown cells was made at 24 and 48 h. MTT (stock: 5 mg/mL in 1X PBS) was added to the wells to establish a final concentration of 0.25 mg/mL. The plates were then incubated at 37 °C for 2 h. Media along with the non-reduced dye was aspirated from all wells, and replaced with DMSO (Fisher Scientific, Hampton, NH). The plates were shaken for 20 min to dissolve the reduced MTT formazan crystals. The absorbance was read at 590 nm using a SpectraMax M2 microplate reader (Molecular Devices, San Jose, CA). Similarly, for assessing HS-5 cell proliferation after exposure to prostate cancer CCM, control and GPC-1 knockdown HS-5 cells were plated in 48-well plates. After an overnight incubation, cells were washed twice with 1X PBS, and treated with either prostate cancer (DU-145 and PC-3) CCM for 24 h and 48 h. CCM was replaced with fresh CCM every 24 h. MTT assays were performed at 24 h and 48 h. For determining PC-3 cell proliferation after exposure to HS-5 CCM, PC-3 cells (2.5 × 10^4^ cells/well in 400 μL complete media) were seeded in 48-well plates. After an overnight incubation, PC-3 cells were exposed to CCM isolated from control and GPC-1 knockdown HS-5 cells for 24 and 48 h, followed by analysis of MTT staining.

### Gel contraction assay

The collagen gel contraction assay was used to assess contraction of HS-5 cells after exposure to prostate cancer CCM or after inhibition of GPC-1. Cells (3 × 10^5^ in 400 μL DMEM) were mixed with 200 μL of collagen I, rat tail stock (3 mg/mL in acetic acid, Invitrogen, Carlsbad, CA) in a 1.5 mL Eppendorf tube. NaOH (1 M) was immediately added (5–6 μL), without any delay, and the mixture was gently pipetted up and down three times while taking care to avoid air bubbles. An NaOH collagen gel titration assay was previously performed to determine the minimum volume of NaOH required to solidify the collagen/DMEM mixture to produce a solidified gel at a neutral pH. After the addition of 1 M NaOH, 500 μL of the mixture was transferred immediately to wells in a 24-well plate. The gels were solidified for 20 min at room temperature. DMEM (500 μL) was added to each well, and the gels were carefully dissociated from the wells. Gels were incubated at 37 °C, and images were taken after 24 h. More details regarding the collagen gel contraction protocol are provided in^32^. The area of the gels was analyzed using ImageJ software (U.S. National Institutes of Health, Bethesda, MD). The effect of prostate cancer CCM on wild-type HS-5 cell contraction was determined by pretreating HS-5 cells with CCM for 48 h. Cells were then collected and their number determined using a hemacytometer, and equal number of cells (3 × 10^5^) were used for the collagen gel contraction assay.

### Scratch wound healing assay

Control and GPC-1 knockdown HS-5 cells were seeded in 6-well plates (5 × 10^5^ cells/well) and incubated for 24 h, or until they reached confluence. A uniform scratch, or a “wound gap”, was created on the cell monolayer using 1 mL pipette tips. The migration capability of the cells was monitored over a period of 24 to 48 h, and images were taken using a Nikon Eclipse T*i* inverted microscope (Nikon, Tokyo, Japan). Cells were stained with 1% (v/v) crystal violet at the last time point before imaging. Cell migration rate was measured as the percent gap closure and normalized to control using the ImageJ software. For analyzing HS-5 cell migration after exposure to CCM, wild-type HS-5, or control and GPC-1 knockdown HS-5 cells were incubated in prostate cancer CCM after creating a wound scratch. Cell migration was assessed at 24 and 48 h, and the cells stained with 1% crystal violet after 48 h before imaging. CCM was replaced with fresh CCM every 24 h. A similar procedure was followed for analyzing PC-3 cell migration after exposure to CCM from control and GPC-1 knockdown HS-5 cells.

### Intratibial administration of RM1 cells in mice

All animal procedures were performed according to the protocol approved by the Institutional Animal Care and Use Committee at the Charlie Norwood Veterans Affairs Medical Center, Augusta, Georgia (protocol 19–04–114). The authors confirm that all animal experiments were performed in accordance with the ARRIVE guidelines 2.0. Intra-tibial injection of murine metastatic prostate cancer cells (RM1) in C57BL/6 mice was performed to induce cancer-induced remodeling, as described previously^33^. Briefly, mice were anesthetized, the injection site was shaven and cleaned with Betadine surgical scrub (Fisher Scientific, Hampton, NH). The knee was flexed and a 27G insulin syringe containing cell suspensions for implantation was used to bore a hole into the proximal end of the left tibia followed by injection of cell solution. Mice were injected intra-tibial with RM1 cells (5 × 10^4^) in 15 μL PBS. The contra-lateral right tibiae were injected with PBS alone as a control for injury-induced remodeling of bone. Bones were analyzed on day 16 after RM1 cell injection. Injected tibiae were fixed and decalcified overnight in Cal-Rite solution (Richard Allan Scientific, Thermo Fisher, Waltham, MA). Tissues were then embedded in paraffin, sectioned (5 μm), and subjected to immunohistochemistry.

### Immunohistochemical analyses of mouse tibial sections

Paraffin-embedded mouse tibial sections were de-paraffinized by using xylene (10 min, 2X) followed by washing in a serial dilution of ethyl alcohol (100% EtOH,10 min, 2X; 95% EtOH, 5 min, 1X; 70% EtOH, 5 min, 1X; 50% EtOH, 5 min, 1X, rinse with distilled water). After de-paraffinization sections were treated with 0.1 mol/L sodium citrate (pH 6.2) and Tween-20 to expose the antigenic epitopes followed by DAB staining (Thermo, Grand Island, NY). Endogenous peroxidases were blocked with 0.9% hydrogen peroxide for 10 min, followed by incubation with 1% bovine serum albumin in PBS for 30 min to eliminate nonspecific binding. The tissue sections were incubated with non-immune IgG (negative control) or GPC-1 (primary) antibodies (dilution 1:100) for 1 h (Cat. No. GTX104557 N3C3; GeneTex, Irvine, California), then with biotinylated anti-rabbit secondary antibodies, and the streptavidin-peroxidase complex for 15 min each. For control studies of the antibodies, the sections were treated with non-IgG containing 2% BSA-PBS instead of the primary antibodies and were confirmed to be unstained. The reaction products were visualized using the substrate 3,30-diaminobenzidine-H_2_O_2_. The sections were counterstained with hematoxylin. After washing, slides were mounted with a clear-mount mounting solution. Visualization and imaging were performed using a Keyence BZ-X800 microscope (Keyence America, Itasca, IL). The quantification of GPC-1 positive cells/tissue was performed using ImageJ FIJI software as previously described (PMID: 31867411). The percentage area was used to determine the area containing cells positive for GPC-1.

### Statistical analysis

All experiments were performed with at least three (n = 3) independent repeats with each n representing cells obtained from a separate passage. Results are represented as average of all repeats ± SEM. Data were statistically analyzed using an one-way or two-way analysis of variance (ANOVA) followed by a Tukey’s post-hoc test, as indicated in the figure legends. GraphPad Prism 8 was used for all statistical analysis, and differences with a *p*-value of < 0.05 were considered significant. The statistical significance cut-off ranges are as follows: *p < 0.05, **p < 0.001, ***p < 0.0001.

## Results

### Effect of GPC-1 inhibition on HS-5 cell morphology and nuclear morphology

**Figure 1:**
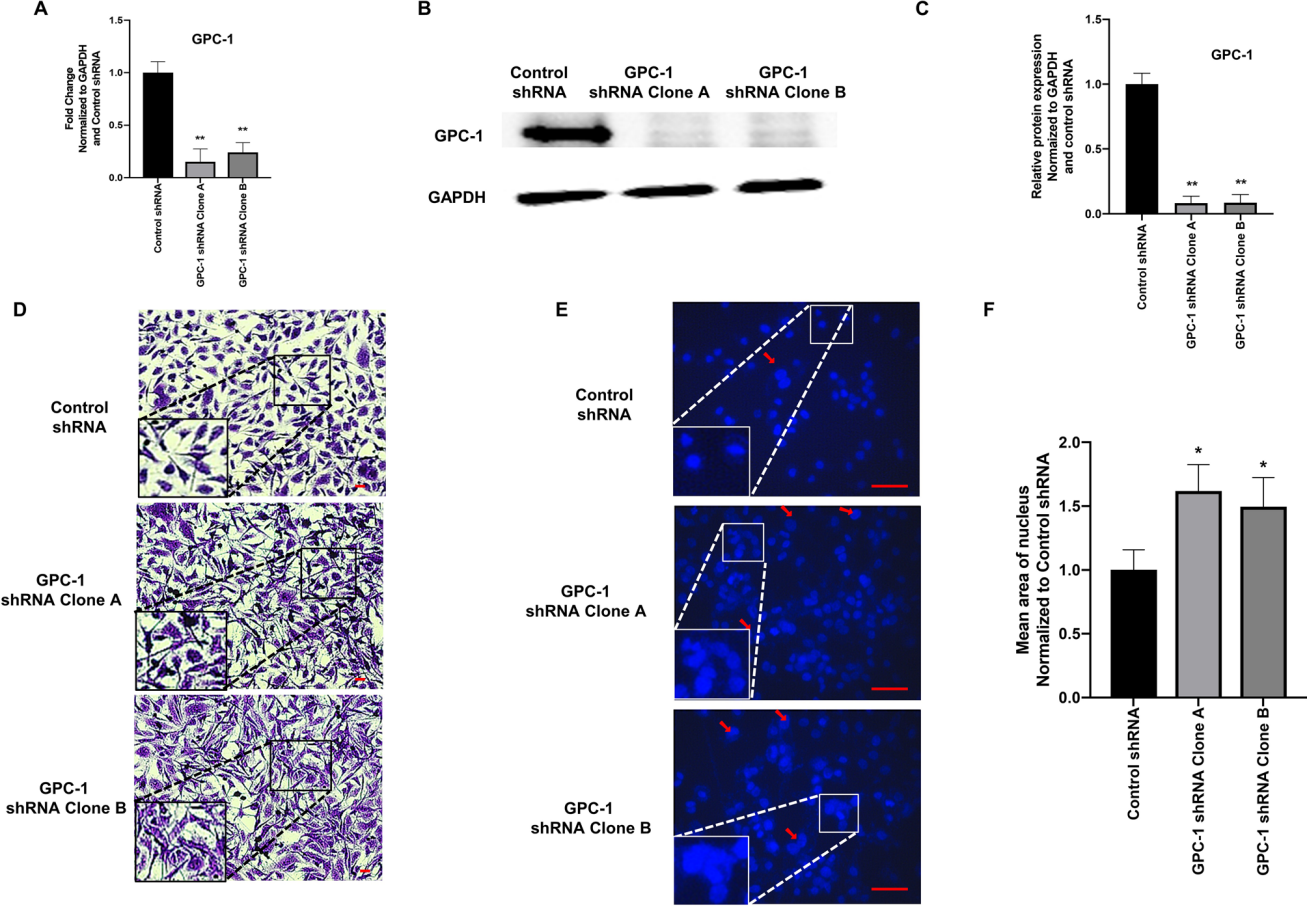
A shRNA-mediated inhibition of GPC-1 mRNA and protein expression in HS-5 cells and its effect on HS-5 cellular and nuclear morphology. (**A)** GPC-1 mRNA and (**B**, **C**) protein expression in HS-5 cells after lentiviral shRNA mediated knockdown of GPC-1, as analyzed by qRT-PCR (**A**) and immunoblot analysis (**B**), followed by protein quantification using densitometric analysis (**C**). GAPDH was used as a housekeeping control. (**D**) Effect of GPC-1 knockdown on HS-5 cell morphology, as visualized by crystal violet staining. (**E**, **F**) Effect of GPC-1 knockdown on HS-5 nuclear morphology, as assessed by DAPI staining (**E**), followed by quantification of nuclear area (**F**). The scale bar in (**D**, **E**) is 5 μm and 50 μm, respectively. Data in (**A**, **C**, **F**) are represented as mean ± SEM. Data are representative of at least three (n = 3) experiments on distinct passages of cells. *p < 0.05 and **p < 0.001 as compared to control using a one-way ANOVA, followed by a Tukey’s post-hoc analysis.

GPC-1 protein expression was attenuated in HS-5 cells using a lentiviral delivery system to test the hypothesis that GPC-1 induces activated fibroblast phenotype in HS-5 cells. Two different GPC-1 shRNA sequences (referred to as clone A and clone B) were used to induce GPC-1 loss, whereas a non-targeting shRNA was used a control. Both clone A and clone B decreased GPC-1 mRNA and protein expression as compared to cells transfected with the control shRNA (Fig. 1A–C). Analysis of morphology revealed that HS-5 cells consisted of two distinct phenotypes, including small spindle-shaped cells and large myofibroblast-like, stellate-shaped cells. However, control shRNA HS-5 cells predominantly consisted of small spindle-like cells. Interestingly, inhibition of GPC-1 increased the relative proportion of stellate-shaped cells, displaying several cytoplasmic extensions (Fig. 1D). No differences were observed in the cellular morphology of wild-type HS-5 cells and control shRNA HS-5 cells, signifying that the lentiviral treatment did not alter the phenotype of HS-5 cells (Supplementary Fig. 2). Assessment of nuclear morphology suggested that GPC-1 inhibition increased the average nuclear size as compared to control (Fig. 1E,F). These data demonstrate that the GPC-1 inhibition alters the cellular and nuclear morphology of HS-5 cells.

### Effect of GPC-1 inhibition on HS-5 cell growth, contractility and migration

**Figure 2:**
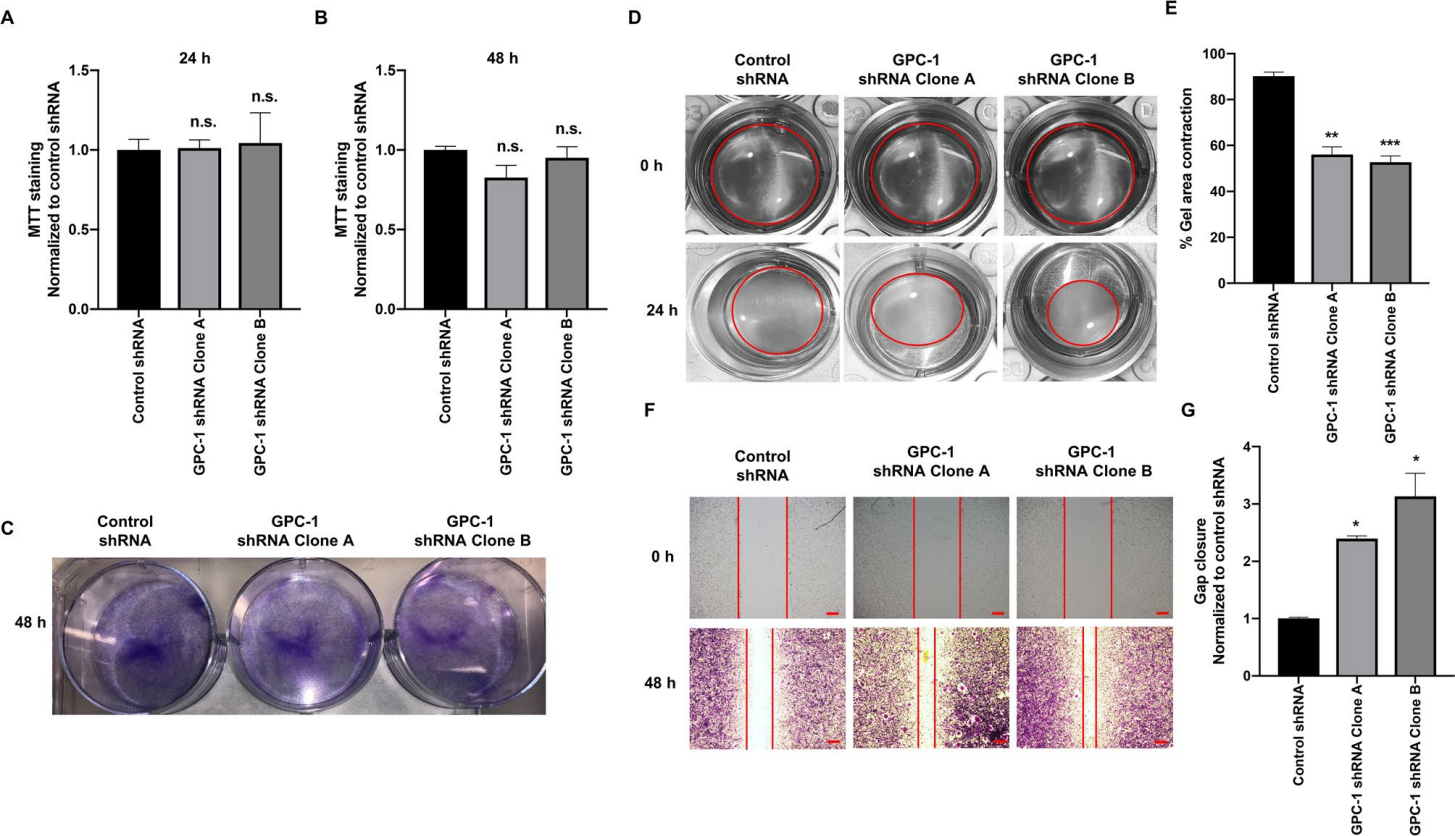
Effect of GPC-1 inhibition on MTT staining, crystal violet staining, contractility and migration in HS-5 cells. (**A**, **B**) Effect of GPC-1 knockdown on MTT staining in HS-5 cells after 24 (**A**) and 48 h (**B**). (**C**) Effect of GPC-1 knockdown on crystal violet staining in HS-5 cells after 48 h. (**D**, **E**) Effect of GPC-1 inhibition on HS-5 cell contraction after 24 h, as determined by the collagen gel contraction assay (**D**), followed by quantification (**E**). (**F**, **G**) Effect of GPC-1 inhibition on HS-5 cell migration after 48 h, as determined by the scratch wound healing assay (**F**), followed by quantification (**G**). The scale bar in (**F**) is 0.05 μm. Data in (**A**, **B**,** E**, **G**) are represented as mean ± SEM. Data are representative of at least three (n = 3) separate experiments on distinct passages. *p < 0.05, **p < 0.001, ***p < 0.0001 and n.s. (not significant) as compared to control using a one-way ANOVA, followed by a Tukey’s post-hoc analysis.

GPC-1 inhibition did not alter significantly the level of MTT staining in HS-5 cells, as compared to control cells, after 24 and 48 h (Fig. 2A,B). Similar results were seen using crystal violet staining (Fig. 2C). These data demonstrate GPC-1 inhibition does not alter HS-5 cell viability or proliferation. As such, the change in HS-5 cell morphology induced by loss of GPC-1 does not appear to be mediated by changes in viability or proliferation.

We also tested the effect of GPC-1 inhibition on HS-5 cell contractility using a 3D collagen gel contraction assay. In agreement with changes in cell morphology, GPC-1 inhibition increased gel contraction in both clones of GPC-1 shRNA HS-5 cells after 24 h, suggesting that the loss of GPC-1 increases contractile properties of HS-5 cells (Fig. 2D,E). In addition, GPC-1 inhibition increased HS-5 cell migration after 48 h, in comparison to controls, as assessed using a scratch wound healing assay (Fig. 2F,G).

### Effect of GPC-1 inhibition on inflammation and CAF marker expression in HS-5 cells

**Figure 3:**
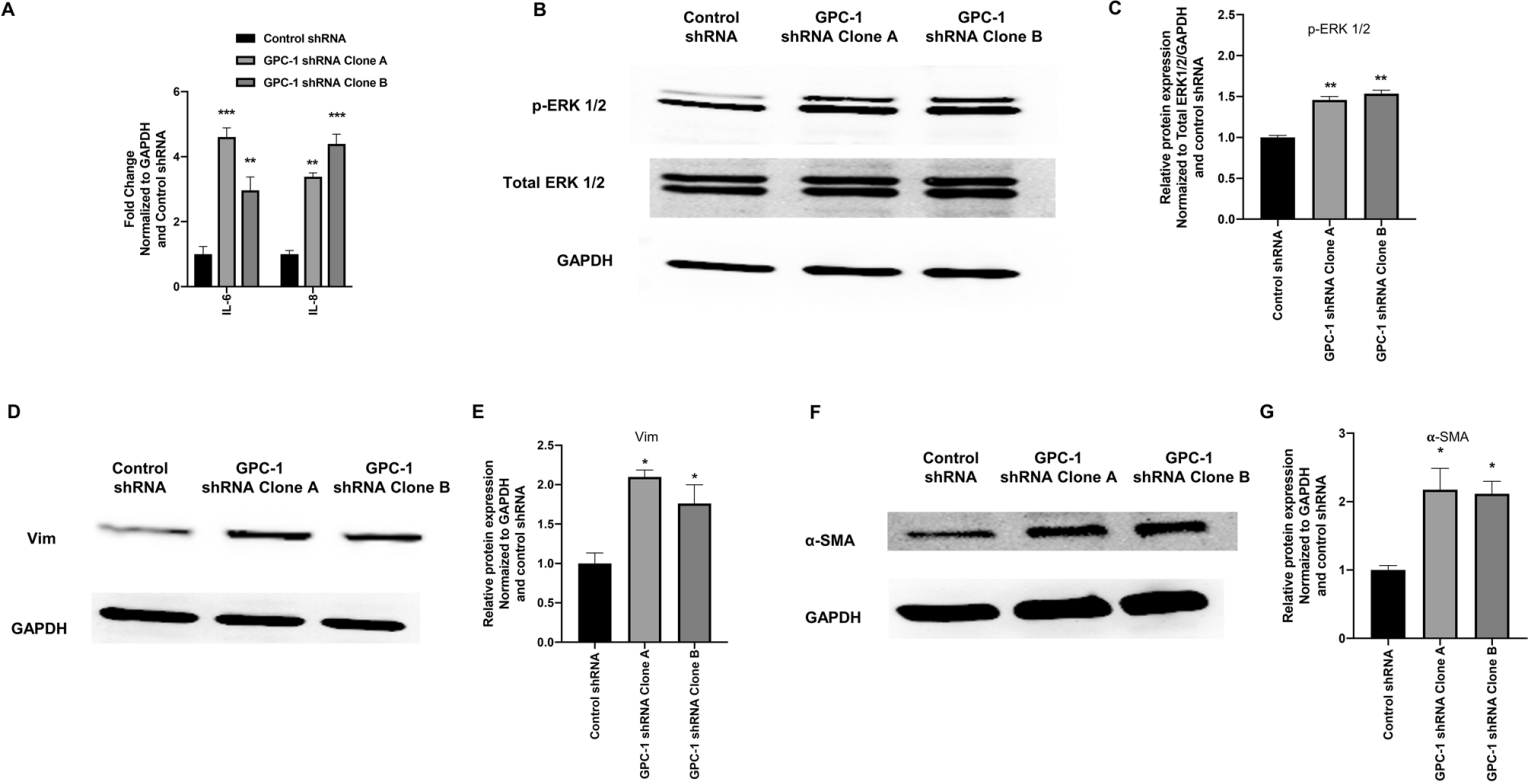
Effect of GPC-1 inhibition on pro-inflammatory and CAF markers in HS-5 cells. (**A**) Effect of GPC-1 inhibition on IL-6 and IL-8 mRNA expression, as analyzed by qRT-PCR. (**B** and **C**) Effect of GPC-1 inhibition on p-ERK 1/2 protein expression in HS-5 cells, as analyzed by immunoblot analysis (**B**), followed by protein quantification using densitometric analysis (**C**). (**D**–**G**) Effect of GPC-1 inhibition on vimentin (Vim) (**D**) and α-smooth muscle actin (α-SMA) (**F**) protein expression in HS-5 cells, as analyzed by immunoblot analysis, followed by protein quantification using densitometric analysis (**E**, **G**). GAPDH was used as a housekeeping control. Data in (**A**, **C**,** E**, **G**) are represented as mean ± SEM. Data are representative of at least three (n = 3) separate experiments on distinct passages.*p < 0.05, **p < 0.001 and ***p < 0.0001 as compared to control using a one-way (**C**, **E**, **G**) or two-way ANOVA (**A**), followed by a Tukey’s post-hoc analysis.

CAFs isolated from various solid cancers display enhanced expression of several inflammatory genes^34^. Therefore, we determined the impact of GPC-1 inhibition in HS-5 cells on the expression of pro-inflammatory cytokines, IL-6 and IL-8 mRNA, and activation of the mitogen activated protein kinase/extracellular signal-regulated kinase (MAPK/ERK) pathway. GPC-1 inhibition in HS-5 cells significantly increased both IL-6 and IL-8 mRNA levels, in comparison to controls after 48 h (Fig. 3A). Similar results were seen when assessing p-ERK 1/2 protein expression using immunoblot analysis (Fig. 3B,C).

We also assessed the effect of GPC-1 inhibition in HS-5 cells on the protein expression of vimentin and α-smooth muscle actin (α-SMA), cytoskeletal proteins differentially expressed in quiescent and activated fibroblasts, including CAFs^2^. GPC-1 inhibition in HS-5 cells significantly increased the expression of vimentin and α-SMA protein, in comparison to control cells (Fig. 3D–G).

### Effect of GPC-1 inhibition on MTT staining, cell morphology and cell migration in HS-5 cells after treatment with prostate cancer CCM

**Figure 4:**
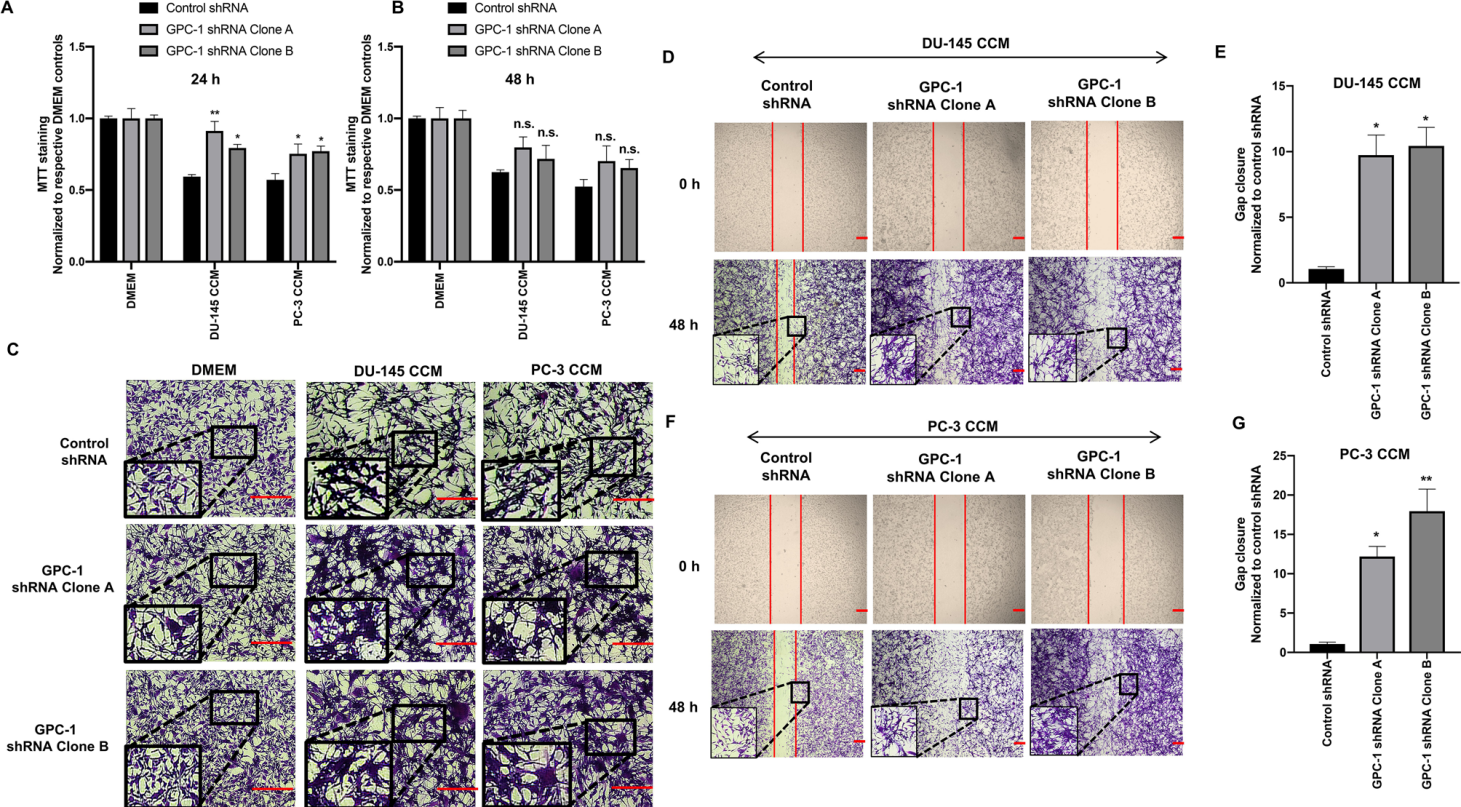
Effect of GPC-1 inhibition on MTT staining, cell morphology and cell migration in HS-5 cells after exposure to prostate cancer cell conditioned media (CCM). (**A**, **B**) Effect of GPC-1 inhibition on MTT staining in HS-5 cells after exposure to DU-145 or PC-3 CCM for 24 (**A**) and 48 h (**B**). (**C**) Effect of GPC-1 inhibition on HS-5 cell morphology after exposure to DU-145 or PC-3 CCM after 24 h, as observed by crystal violet staining. (**D**–**G**) Effect of GPC-1 inhibition on HS-5 cell migration after 48 h exposure to DU-145 CCM (**D**) or PC-3 CCM (**F**), as determined by the scratch wound healing assay, followed by quantification (**E**, **G**). The scale bar in (**C**, **D**, **F**) is 0.05 μm. Data in (**A**, **B**, **E**, **G**) are represented as the mean ± SEM. Data are representative of at least three (n = 3) separate experiments. *p < 0.05, **p < 0.001 and n.s. (not significant) as compared to control using a one-way ANOVA, followed by a Tukey’s post-hoc analysis.

The effect of GPC-1 inhibition on MTT staining in HS-5 cells when exposed to CCM from prostate cancer cells was determined. Exposure of control HS-5 cells (those transfected with scrambled shRNA) with CCM from DU-145 or PC-3 cells reduced MTT staining after 24 h (Fig. 4A). Similar results were obtained when wild-type HS-5 cells were treated with prostate cancer CCM (Supplementary Fig. 3A), suggesting that the reduction in MTT staining upon treatment with CCM was not altered by transfection of shRNA.

Inhibition of GPC-1 in HS-5 cells prior to exposure to CCM from either PC-3 or DU-145 cells significantly increased MTT staining after 24 h, as compared to control shRNA HS-5 cells (Fig. 4A). A similar trend was observed at 48 h, but the data was not significant (Fig. 4B). Exposure of HS-5 cells with CCM from prostate cancer cells also induced significant changes in cell morphology after 24 h. Specifically, inhibition of GPC-1 resulted in a more aggressive phenotype in HS-5 cells (Fig. 4C), which was enhanced when these cells were exposed to CCM from either DU-145 or PC-3 cells. The changes included structural changes and increased amounts of dense net of cells and cell aggregates.

The effect of GPC-1 inhibition on HS-5 cell migration after exposure to prostate cancer CCM was analyzed using a scratch wound healing assay. GPC-1 inhibition in HS-5 cells increased gap closure after exposure to CCM from either DU-145 or PC-3 cells after 48 h, as compared to control HS-5 cells (Fig. 4D–G), indicating that GPC-1 inhibition in HS-5 cells increased their migration when exposed to prostate cancer CCM. Furthermore, HS-5 cells exposed to CCM exhibited collective migration towards the wound gap, indicating these cells migrated as cohorts instead of single cells. No significant change in wound closure was observed in wild-type HS-5 cells after treatment with prostate cancer CCM (Supplementary Fig. 3B,C).

### Effect of GPC-1 inhibition in HS-5 cells on PC-3 cell morphology, MTT staining and cell migration and gene expression

**Figure 5:**
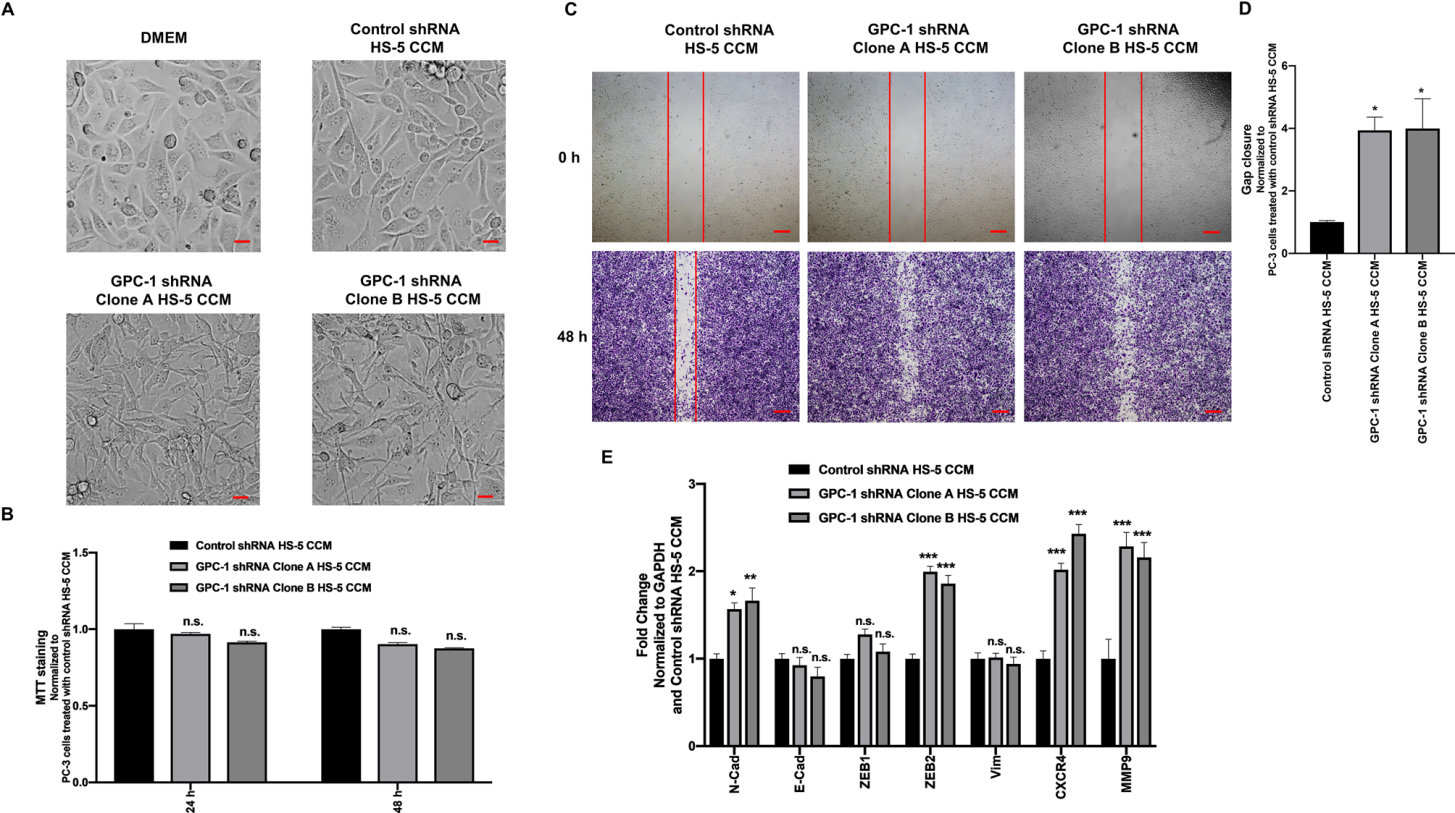
Effect of GPC-1 knockdown HS-5 CCM on PC-3 cell morphology, MTT staining, cell migration and gene expression. (**A**) PC-3 cell morphology after a 24 h exposure to DMEM or CCM from control shRNA HS-5, or clone A and clone B GPC-1 shRNA HS-5 cells. (**B**) MTT staining in PC-3 cells after exposure to CCM from control shRNA HS-5, or clone A and clone B GPC-1 shRNA HS-5 cells for 24 and 48 h. (**C**, **D**) PC-3 cell migration after exposure to CCM from control shRNA HS-5 cells, or clone A and clone B GPC-1 shRNA HS-5 cells for 48 h, as determined by a scratch wound healing assay (**C**), followed by quantification (**D**). (**E**) mRNA expression of EMT marker genes in PC-3 cells after exposure to CCM from control shRNA HS-5, or clone A and clone B GPC-1 shRNA HS-5 cells for 24 h, as determined by qRT-PCR. GAPDH was used as the housekeeping control and data was normalized to PC-3 cells treated with control shRNA HS-5 CCM. The scale bar in (**A**, **C**) is 5 μm and 0.05 μm, respectively. Data are represented as mean ± SEM. Data are derived from at least three (n = 3) independent passages. *p < 0.05, **p < 0.001, ***p < 0.0001 and n.s. (not significant) as compared to control using a one-way (**B**, **D**) or two-way (**E**) ANOVA, followed by a Tukey’s post-hoc analysis.

To investigate the effect of GPC-1 inhibition in HS-5 cells on prostate cancer cell morphology, we exposed PC-3 cells with CCM isolated from HS-5 cells. CCM isolated from HS-5 cells transduced with control shRNA showed only slight changes in cell morphology as compared to control cells (Fig. 5A). In contrast, PC-3 cells exposed to CCM isolated from HS-5 cells in which GPC-1 expression was inhibited had significant changes in cell morphology after 24 h (Fig. 5A), which included a change from an epithelial-to-mesenchymal-like morphology, along with a reduced cell-to-cell contact. Exposed PC-3 cells also showed a spindle-shaped phenotype.

The functional effect of GPC-1 on cell growth and proliferation was tested by exposing PC-3 cells to CCM isolated from HS-5 cells in which GPC-1 was inhibited and then assessing MTT staining. No significant differences were observed in MTT staining in PC-3 cells exposed to HS-5 CCM after GPC-1 inhibition after 24 and 48 h (Fig. 5B). These data suggest that GPC-1 is not involved in increasing prostate cancer proliferation by paracrine stimulation in the TME.

We also determined if GPC-1 inhibition in HS-5 cells affects PC-3 cell migration. As such, we exposed PC-3 cells to HS-5 CCM and assessed cell migration using a scratch wound healing assay. Exposure of PC-3 cells to CCM isolated from HS-5 cells in which GPC-1 was inhibited increased the percentage of wound healing as compared to PC-3 cells exposed to CCM isolated from control HS-5 cells (Fig. 5C,D). These data suggest that inhibition of GPC-1 in HS-5 cells alters the effect of CCM on prostate cancer cell migration.

The above data suggest that inhibition of GPC-1 in HS-5 cells induces paracrine activation in PC-3 cells. We tested this hypothesis by determining the expression of several markers of epithelial-to-mesenchymal transition (EMT) and prostate cancer aggression and metastasis^35–37^ in PC-3 cells after exposure to HS-5 CCM. As expected, exposure of PC-3 cells with CCM isolated from HS-5 cells in which GPC-1 was inhibited significantly increased the level of mRNA for N-Cad, ZEB2, CXCR4 and MMP9 after 24 h, as compared to those cells exposed to CCM isolated from control HS-5 cells (Fig. 5E). Interestingly, there were no significant changes in mRNA expression of E-Cad, Zeb-1 or vimentin, suggesting that the effect of GPC-1 on EMT was only partial.

### GPC-1 expression in the bone of a metastatic prostate cancer murine model following bone remodeling

**Figure 6:**
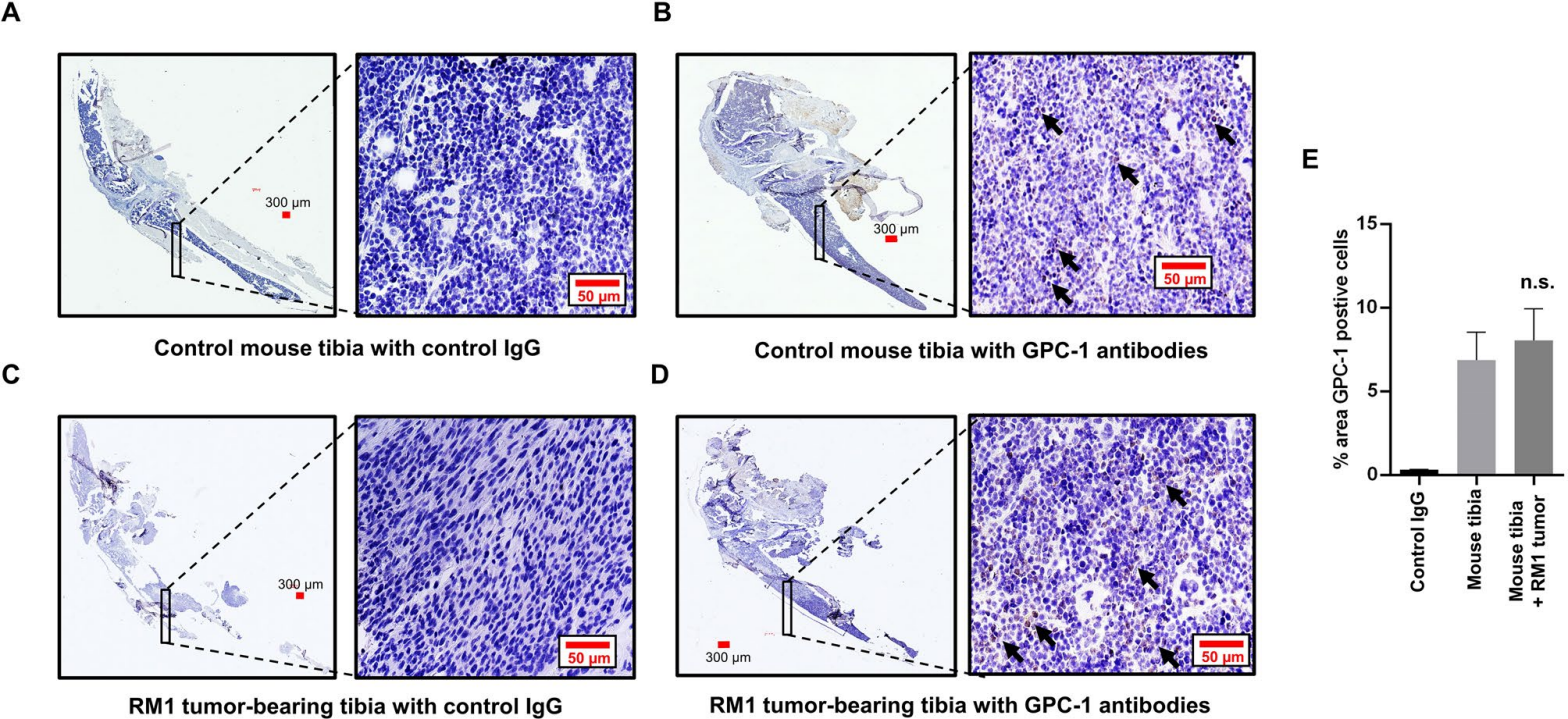
Bone expression of GPC-1 in the murine metastatic prostate cancer bone model. (**A**) Control mouse tibia probed with non-immune antibodies, stained by DAB and counter-stained by H&E. (**B**) Control mouse tibia probed with GPC-1 antibodies, stained by DAB and counter-stained by H&E. (**C**) Prostate tumor cell bearing tibia probed with non-immune antibodies, stained by DAB and counter-stained by H&E. (**D**) Prostate tumor cell bearing tibia probed with GPC-1 antibodies, stained by DAB and counter-stained by H&E. Arrows in (**B**, **D)** indicate GPC-1 positive cells. (**E**) Quantification of percent area of bone stained positive for GPC-1 as determined using Image J FIJI statistical software. The scale bar in (**A**–**D**) is 50 μm. Data are presented as the mean ± SEM. Data in (**A**–**D**) is representative of at least 6 separate mice (n = 6). n.s. (not significant) as compared to control mouse tibia using an unpaired Student’s t-test.

A limitation of the above data is the it was generated using cells lines. We addressed this limitation by using a tumor model involving intra-tibial injection of murine metastatic prostate cancer cells (RM1) in C57BL/6 mice, which was used to induce cancer-induced remodeling, primary bone loss, as described in our recent study ^33^. As previously reported^33^, injection of RM1 cells into mouse tibia induced significant bone loss (Fig. 6A,C). Assessment of GPC-1 staining in control mouse tibia (those not receiving RM1 cells) demonstrated several positive cells, especially in the long bone portion, but also in the parenchyma (Fig. 6B, arrows). Injection of RM1 cells into the tibia did appear to decrease GPC-1 positive cells (Fig. 6D), but this was primarily due to loss of bone, as the number of cells staining positive for GPC-1 were similar in both control tibia and those injected with RM1 cells (Fig. 6E). These data demonstrate that GPC-1 is expressed in bone cells and is still present during bone loss induced by prostate cancer cells.

## Discussion

The ability of glypicans to regulate fibroblast behavior and their transformation into CAFs has not been previously investigated. Our recent study showed that GPC-1 acts as a tumor suppressor in prostate cancer as the loss of GPC-1 induced a reactive TME in vivo^28^. This study also showed that human foreskin fibroblasts exposed to conditioned media from prostate cancer cells in which GPC-1 was inhibited exhibited an increase in mRNA expression of several genes involved in fibroblast activation. These data suggest that GPC-1 plays a paracrine role in prostate cancer progression by facilitating tumor-stromal interaction^28^. Furthermore, a study by Suhovskih et al. showed that the expression of GPC-1 mRNA was reduced in immortalized human fibroblasts upon co-culture with human prostate cancer cell-lines^38^. Moreover, treatment of human lung fibroblasts with TGF-β, which is an inducer of fibroblast activation, also reduced GPC-1 protein mRNA and protein expression^39^. The above studies support the hypothesis that GPC-1 mediates fibroblast behavior, a hypothesis previously suggested by others; however, to date, no studies directly determined the effect of GPC-1 inhibition on fibroblast activation. To the best of our knowledge, data reported in our study are among the first to demonstrate the significance of GPC-1 loss in the induction of activated fibroblast-like behavior in BSCs in the tumor stroma.

It is to be noted that our initial studies demonstrated an increase in GPC-1 expression in HS-5 cells following an acute exposure to prostate cancer CCM (Supplementary Fig. 4A). It is possible that exposure of HS-5 cells to prostate cancer CCM induced a compensatory mechanism to retain GPC-1 in HS-5 cells to prevent BSC switch to activated fibroblasts. GPC-1 expression has been previously reported to be modulated by DNA hypomethylation^40^ and microRNA-149^41^. These mechanisms could have played a role in increasing the acute GPC-1 expression in HS-5 cells following treatment with prostate cancer CCM.

One of the defining characteristics of fibroblast activation are phenotypic changes, including changes in cell and nuclear morphology. This morphological switching entails a transition from a spindle shape to a stellate-like shape^2,7^. The change in HS-5 cell and nuclear morphology induced by GPC-1 inhibition is similar to that observed in CAFs, with large, relatively plump cells possessing long cytoplasmic extensions. GPC-1-dependent phenotypic changes were similar to that seen in wild type HS-5 cells when exposed to CCM from prostate cancer cells (Supplementary Fig. 4B). HSPGs, such as GPC-1, regulate cell shape and size^42,43^. Therefore, it is not surprising to see that GPC-1 regulates HS-5 cell morphology. The effect of GPC-1 inhibition on HS-5 nuclear morphology is consistent with studies in the literature demonstrating that CAFs exhibit relatively larger nuclei, owing to the their euchromatic state and increased transcriptional activity^44^.

CAFs have an increased proliferation rate as compared to resting fibroblasts^45,46^. In our study, GPC-1 inhibition did not alter the inherent proliferative capacity of HS-5 cells. CAFs also possess enhanced contractile properties due to reorganization of stress fibers and alteration of biomechanical pressures exerted by ECM on cells^47^. Our data suggest that GPC-1 modulates the contractile properties of BSCs, which is akin to that observed in CAFs. This is also similar to the behavior of HS-5 cells when they were exposed to prostate cancer CCM, where treated HS-5 cells demonstrated increased collagen gel contraction as compared to control cells (Supplementary Fig. 4C,D). Our finding that GPC-1 modulates HS-5 cell contractility is significant as increased contractility of CAFs enables them to form ECM tracks, which are used by cancer cells for collective migration and invasion^3,46^. Interaction of GPC-1 with collagen chains present in the ECM is one of the plausible mechanisms by which GPC-1 could be modulating ECM stiffness, which, in turn, regulates cell contractility by altering biomechanical pressures exerted by the ECM^24–26^. CAFs also have increased migratory capacity, which is essential for their recruitment to the site of primary and secondary tumor growth^2^. Soluble factors such as cytokines, CXCL12, and growth factors present in the TME are involved in the increased migratory behavior of CAFs. The ability of GPC-1 inhibition to increase HS-5 cell migration may be linked to its ability to modulate the activity of cytokines and growth factors by acting as a co-receptor, by stabilization of ligand-receptor complexes, or by altering ligand availability^15^.

The effect of GPC-1 on of IL-6, IL-8 and p-ERK1/2 expression supports the hypothesis that the loss of GPC-1 triggers a pro-inflammatory phenotype in HS-5 cells. A similar increase in p-ERK 1/2 expression also happens when HS-5 cells are treated with prostate cancer CCM (Supplementary Fig. 4E). Therefore, the loss of GPC-1 mimics the same HS-5 cell pro-inflammatory signature that is observed when they are exposed to prostate cancer CCM. CAFs, being one of the most abundant cell populations in the TME, are important contributors of the tumor-enhancing inflammation. CAFs isolated from initial stages of neoplasm progression possess pro-inflammatory properties^34^. Cytokines, including IL-6 and IL-8, released by CAFs can directly promote inflammation^48^ or indirectly participate in immunomodulation by cytokine-dependent recruitment of immune cells into the TME^34^. Hence, our finding that GPC-1 regulates the inflammatory state of HS-5 cells is fascinating as activated fibroblasts have been touted as novel targets for resolving inflammation in cancer and other inflammatory diseases^49^.

Modulation of p-ERK 1/2 protein levels by GPC-1 has been previously seen in the literature where attenuation of GPC-1 expression is observed with the concomitant reduction in p-ERK 1/2 activation in pancreatic cancer cells^50^. This effect is opposite to the increase in p-ERK 1/2 observed in HS-5 cells upon GPC-1 reduction in our study. This finding is not surprising in terms of the activity of glypicans, which possess dual roles as inhibitors and activators of same pathways in a cell- and tissue-type dependent manner^26^. Vimentin and α-SMA are mesenchymal markers that are frequently upregulated in CAFs^44^. Increased expression of vimentin and α-SMA seen in HS-5 cells following ablation of GPC-1 supports the conclusion that GPC-1 inhibits the switch of BSCs to activated fibroblasts. Basically, the loss of GPC-1 recapitulated several intrinsic features of CAF behavior in HS-5 cells.

As the loss of GPC-1 recapitulated several intrinsic features of CAF behavior in HS-5 cells, we next determined how GPC-1 regulates the phenotypic and functional behavior of HS-5 cells when exposed to a model of the prostate cancer TME in the form of CCM. Activation of fibroblast induces a myofibroblast-like phenotype, along with increased cell aggregation or cell syncytia due to their altered ability to adhere to each other^51^. The increased amount of aggregation in cells in which GPC-1 was inhibited supports the conclusion that this protein mediates aggregation, at least in HS-5 cells after exposure to CCM. Heparan sulfate (HS) chains are reported to be negative regulators of cell syncytia formation, and cell–cell fusion^52^. Based on our preliminary data showing GPC-1 mRNA to be the most abundant HSPG of the glypican family in HS-5 cells (Supplementary Fig. 5A), we hypothesized that GPC-1 is a significant source of HS chains in HS-5 cells. This hypothesis, if proven true, would suggest that the loss of GPC-1 leads to an overall reduction of HS chains in HS-5 cells. This loss would then facilitate increased cell–cell fusion, which allows for increased cell aggregation after exposure to CCM. Regardless of the mechanisms involved, our study is the first to report that GPC-1 modulates cell aggregation in BSCs after their exposure to prostate cancer CCM.

CAFs also display increased proliferation rates^2^. While wild-type HS-5 cells did not increase their proliferation after exposure to CCM, inhibition of GPC-1 did increase MTT staining in comparison to controls cells. CCM isolated from prostate cancer cells was more depleted in nutrients as compared to control (DMEM), and hence, could have led to reduced proliferation of wild-type HS-5 cells. The reduction in proliferation of HS-5 cells after exposure to prostate cancer CCM agrees with similar reports in the literature showing reductions in fibroblast proliferation rates after treatment with prostate cancer CCM^53,54^. However, the increase in proliferation after exposure to CCM in cells in which GPC-1 was inhibited is a novel finding. The increase in MTT staining could be a result of metabolic reprogramming and recycling of available nutrients, which are a hallmark of CAFs^2^.

CAFs migrate as cohesive units due to their altered cell-adhesion behavior in the TME^51^. This agrees with the observation in our study, where we saw increased migration of HS-5 cell aggregates following GPC-1 loss when treated with prostate cancer CCM. Collective migration of CAFs helps in exerting appropriate mechanical pressures in the TME and plays an important role in the remodeling of the ECM. Hence, our finding that GPC-1 alters the collective migration of HS-5 cells is significant as it proposes a novel function of GPC-1 in the modulation of the TME via alteration of CAF behavior. The role of GPC-1 is further supported by the fact that no significant change was seen in the migration of wild-type HS-5 cells after treatment with prostate cancer CCM (Supplementary Fig. 3B,C). GPC-1 inhibition increased the inherent migratory capacity of HS-5 cells. However, migration rates were further increased, and the cells migrated as a cohort when treated with prostate cancer CCM after GPC-1 inhibition. These data support the hypothesis that GPC-1 acts to inhibit BSC migration, which may be a mechanism for tumor suppression.

While these data were derived using multiple prostate cancer cells, they are limited in that they were derived from a single cell model. Thus, studies are needed using stromal cells from other sources, such as those isolated from primary and secondary tumors and other organ sites. Such studies will corroborate the results of this study, and help strengthen the role of GPC-1 as a regulator of fibroblast activation in the TME. We partially addressed this limitation using a human foreskin-derived fibroblast cell-line which had not been genetically modified (HS-27 cells). Interestingly, HS-27 cells have higher levels of GPC-1 expression as compared to HS-5 cells (Supplementary Fig. 5B), with a differential expression of CAF markers, α-SMA and vimentin (Supplementary Fig. 5C). Future studies comparing the role of GPC-1 in modulating fibroblast activation in these cell-lines can provide useful insights into the functions of GPC-1 as a modulator of fibroblast behavior in the TME.

Data in this study support the conclusion that the exposure of prostate cancer cells to CCM from HS-5 cells in which GPC-1 was inhibited increases the acquisition of a mesenchymal phenotype and increases the rate of cell migration after wound healing. This suggests that GPC-1 mediates the ability of activated HS-5 cells to alter PC-3 cell morphology and migration. CAFs also promote prostate cancer cell proliferation. However, in our study, we did not see any changes in the PC-3 cell proliferation following treatment with HS-5 CCM after GPC-1 inhibition, suggesting that GPC-1 plays a selective role in the paracrine activation of prostate cancer cells in the bone microenvironment. This aligns with the fact that inhibition of GPC-1 did not alter all markers of EMT in PC-3 cells exposed to CCM. Our finding that several markers of EMT and prostate cancer aggressiveness are elevated in PC-3 cells following treatment with CCM collected from HS-5 cells exhibiting GPC-1 loss further supports these observations. Together, these data suggest that GPC-1 indirectly regulates prostate cancer phenotype, migratory behavior and gene expression via the induction of activated fibroblasts in the bone microenvironment.

There is very few studies assessing the expression of GPC-1, or any glypican for that matter, in bone cells in prostate cancer models. Our studies demonstrate the expression of GPC-1 in bone cells present in both control mice tibia as well as those undergoing bone loss induced by RM1 cells injection. While the overall level of GPC-1 did appear to decrease between the two models, it’s important to point out that the model used cannot easily differentiate between different types of cells. Nevertheless, these data do demonstrate that GPC-1 is present in bone cells. Further, studies are needed to determine the specific cell types and assess overall protein expression levels, as well as the effect of genetic manipulation of GPC-1 expression on bone remodeling induced by prostate cancer.

Collectively, our data suggest that in addition to supporting the intrinsic phenotypic characteristics in BSCs, GPC-1 also regulates their extrinsic phenotype to support the activated fibroblasts’ ability to increase prostate cancer cell migration and EMT. The increase in paracrine activation of markers of cancer progressiveness in prostate cancer cells and the change their in cell morphology, following exposure to HS-5 cells in which GPC-1 was inhibited suggests that GPC-1 loss indirectly promotes an aggressive prostate cancer phenotype. This supports the hypothesis that GPC-1 acts as an inhibitor of prostate tumor growth, an observation supported by our recent in vivo studies^28^. Hence, our novel findings regarding the functions of GPC-1 in managing the intrinsic and extrinsic CAF phenotypes of BSCs in prostate cancer are important as they suggest GPC-1 as a novel anti-CAF target in prostate cancer.

## Supplementary Information

Supplementary Information.

## Data Availability

The datasets generated during and/or analyzed during the current study are available from the corresponding author on reasonable requests.

## Abbreviations

α-SMA: α-Smooth muscle actin
BSCs: Bone marrow-derived stromal cells
CAFs: Cancer associated fibroblasts
CCM: Cell conditioned media
DAPI: 4′,6-Diamidino-2-phenylindole
ECM: Extracellular matrix
EMT: Epithelial-to-mesenchymal transition
ERK: Extracellular signal-regulated kinase
FBS: Fetal bovine serum
GEPIA: Gene expression profiling interactive analysis
GPC-1: Glypican-1
GPI: Glycosylphosphatidylinositol
HSPGs: Heparan sulfate proteoglycans
IL: Interleukin
MAPK: Mitogen activated protein kinase
MSCs: Mesenchymal stem cells
MTT: 3-(4,5-Dimethylthiazolyl-2)-2,5-diphenyltetrazolium bromide
qRT-PCR: Quantitative real-time polymerase chain reaction
TME: Tumor microenvironment
Vim: Vimentin
