# Supplementary material for: Inhibition of glypican-1 expression induces an activated fibroblast phenotype in a human bone marrow-derived stromal cell-line
Source: Sci Rep. 2021 Apr 29;11:9262. doi: 10.1038/s41598-021-88519-7 (PMC8084937; doi:10.1038/s41598-021-88519-7)
Supplement: Supplementary file 1 — Supplementary Information. [file 41598_2021_88519_MOESM1_ESM.docx]

**Inhibition of Glypican-1 Expression Induces an Activated Fibroblast Phenotype in a Human Bone Marrow-Derived Stromal Cell-line**​

Sukhneeraj P. Kaur1, Arti Verma2, Hee. K. Lee3, Lillie M. Barnett4, Payaningal R. Somanath2,5  and Brian S. Cummings1,4*​

1Department of Pharmaceutical and Biomedical Sciences, College of Pharmacy, University of Georgia, Athens, GA, USA​

2Clinical and Experimental Therapeutics, University of Georgia and Charlie Norwood VA Medical Center, Augusta, GA, USA​

3Medical College of Georgia, Augusta University, Augusta, GA, USA ​

4Interdisciplinary Toxicology Program, University of Georgia, Athens, GA, USA ​

5Department of Medicine and Vascular Biology Center, Augusta University, Augusta, GA, USA  ​

*Corresponding Author​

450 College of Pharmacy South​

University of Georgia​

Athens, GA 30607​

Phone: 706-542-3792​

Fax: 706-542-5358​

E-Mail: [briansc@uga.edu](mailto:briansc@uga.edu) ​

**Supplementary Figure Legends**

**
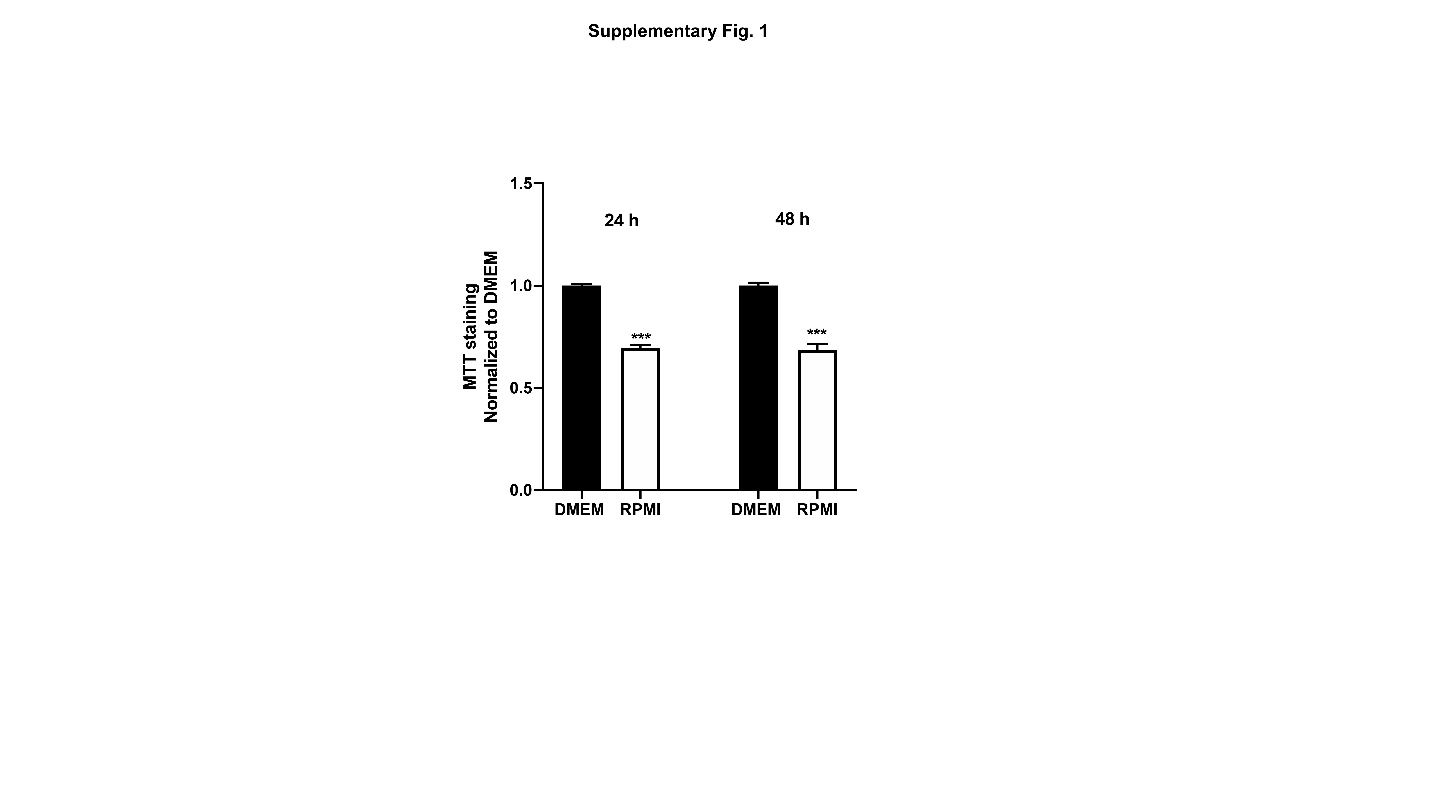
**

**Supplementary Fig. 1: Effect of DMEM and RPMI media on MTT staining in wild-type HS-5 cells.** Effect of serum-free DMEM and RPMI media on MTT staining of wild-type HS-5 cells after 24 and 48 h. DMEM was used as control. Data are represented as mean ± SEM. Data are derived from at least three (n = 3) independent passages. ****p* < 0.0001 as compared to control using a one-way ANOVA, followed by a Tukey’s post-hoc analysis.


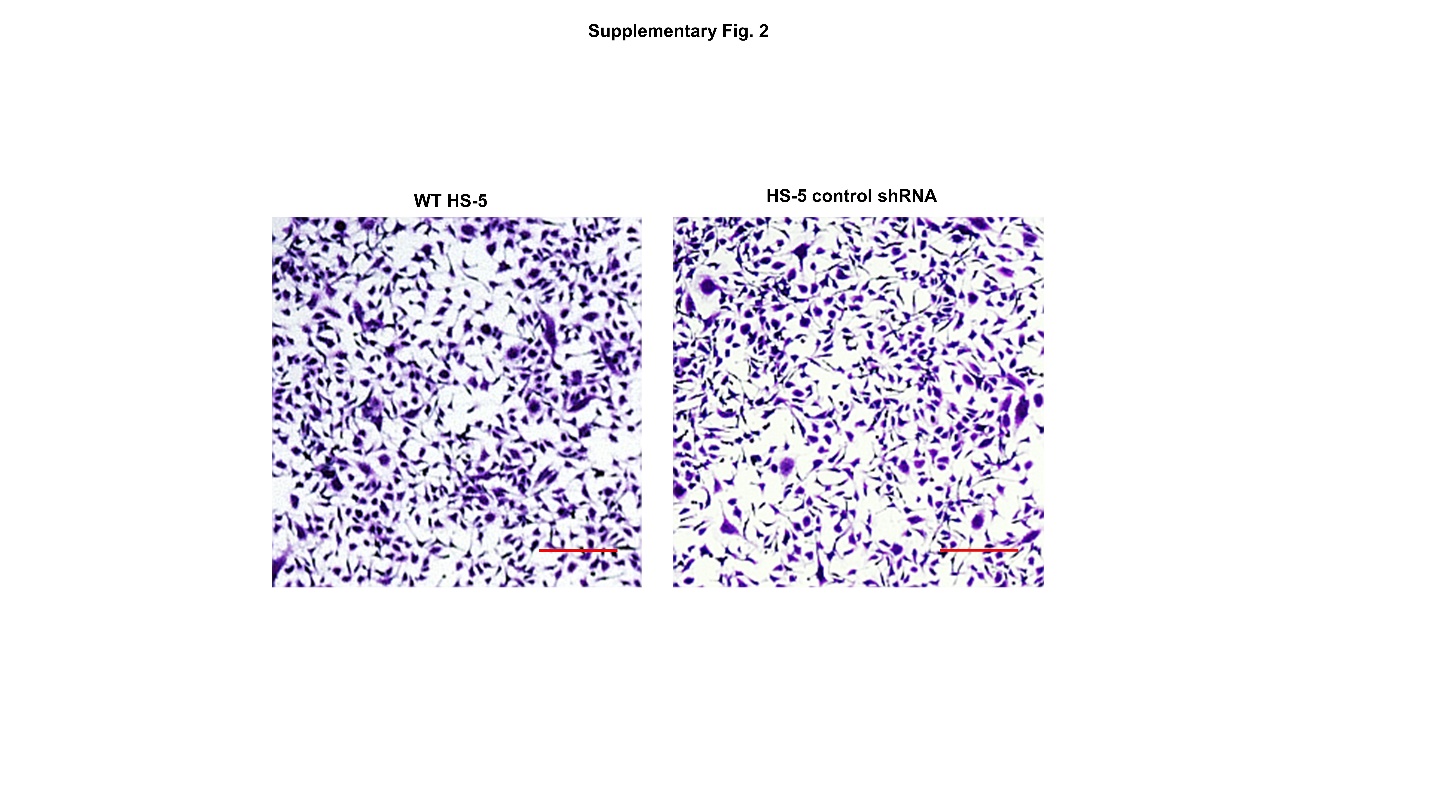


**Supplementary Fig. 2 : Cellular morphology of wild-type HS-5 and control shRNA-treated HS-5 cells.** Cell morphology of wild-type HS-5 and control shRNA HS-5 cells, as visualized by crystal violet staining. The scale bar is 0.05 μm.


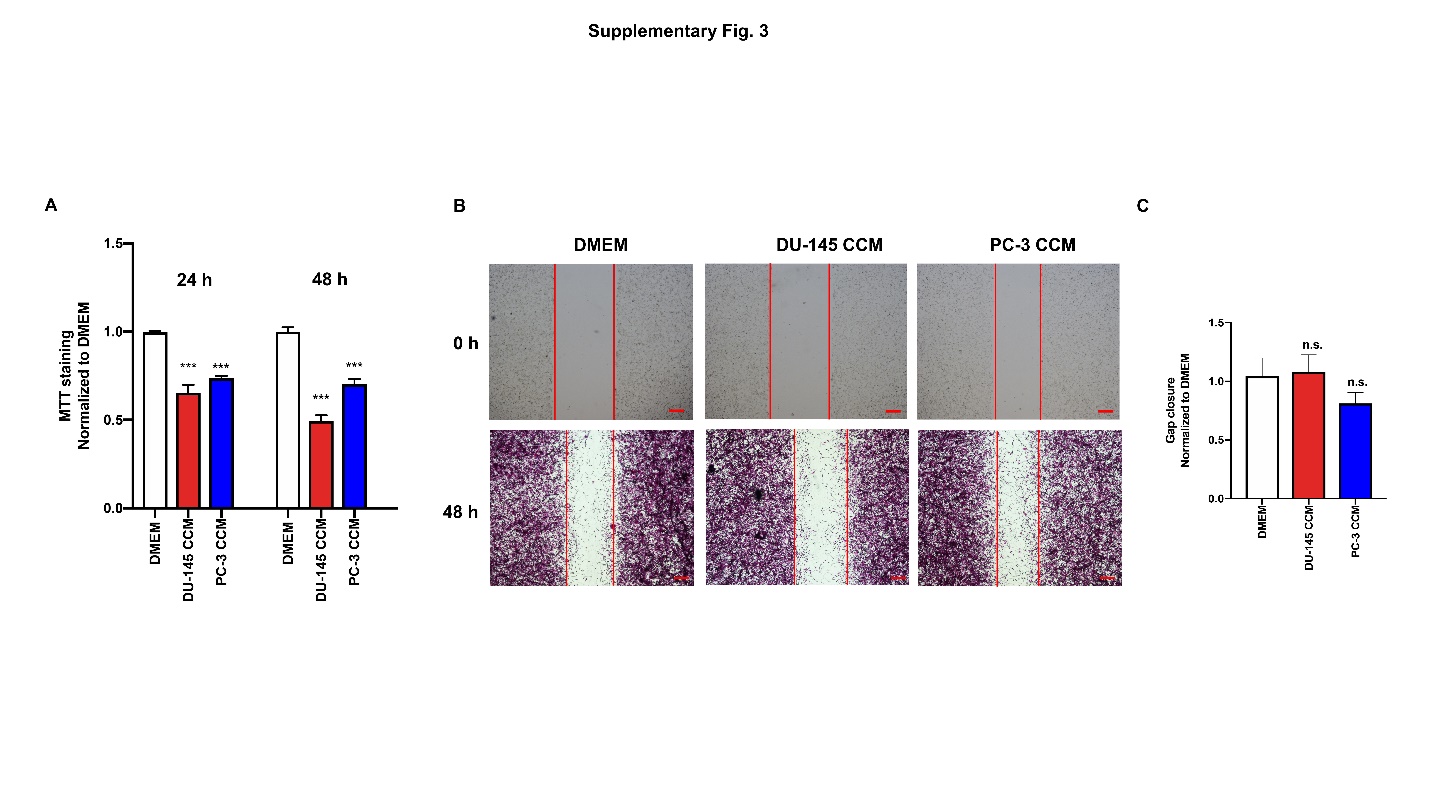


**Supplementary Fig.3: Effect of prostate CCM on MTT staining and cell migration in wild-type HS-5 cells:** (**A**) Effect of DU-145 CCM and PC-3 CCM on MTT staining in wild-type HS-5 cells at 24 and 48 h. (**B, C**) Effect of DU-145 CCM and PC-3 CCM (**B**) on wild-type HS-5 cell migration after 48 h, as determined by the scratch wound healing assay, followed by quantification (**C**). The scale bar in (**B**) is 0.05 μm. Data in (**A** and **C**) are represented as mean ± SEM. Data are representative of at least three (n = 3) separate experiments. ****p* < 0.0001 and n.s. (not significant) as compared to control using a one-way ANOVA, followed by a Tukey’s post-hoc analysis.

**
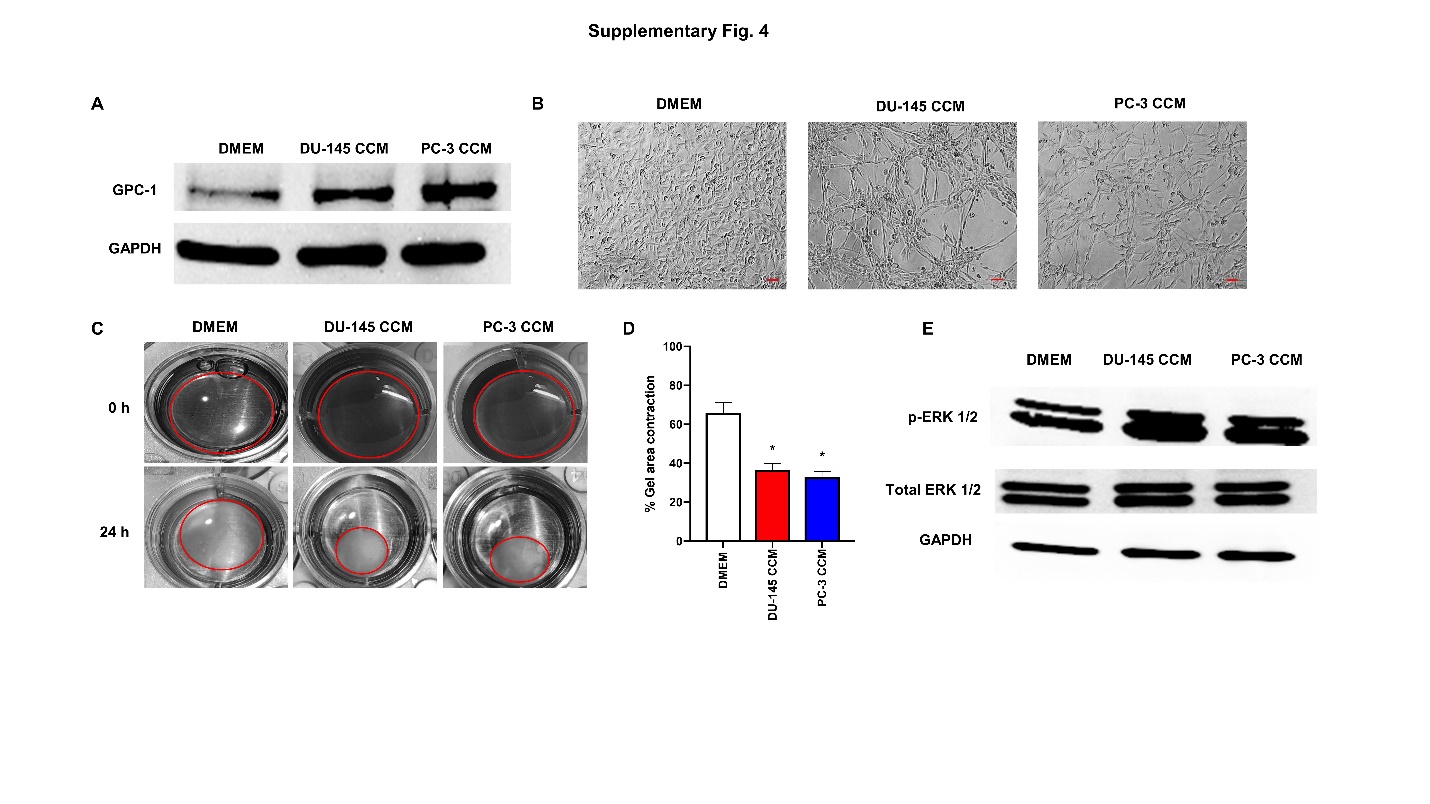
**

**Supplementary Fig. 4: GPC-1 and p-ERK 1/2 protein expression, cell morphology and contraction in wild-type HS-5 cells after exposure to prostate cancer CCM.** (**A**) Effect of DU-145 CCM and PC-3 CCM on GPC-1 protein expression in wild-type HS-5 cells after 48 h, as analyzed by immunoblot analysis. GAPDH was used as a housekeeping control. (**B**) Wild-type HS-5 cells were exposed to DU-145 CCM or PC-3 CCM for 24 h, and the morphology was assessed by phase contrast microscopy. (**C** and **D**) Cell contractility of wild-type HS-5 cells after exposure to prostate cancer CCM. Wild-type HS-5 cells were exposed to DU-145 CCM or PC-3 CCM for 48 h. Cells in all conditions were counted and equal number of cells were embedded in collagen gels for measuring the collagen gel contraction (**C**). The area of the gels were measured after 24 h, followed by their quantification (**D**). (**E**) Effect of DU-145 CCM or PC-3 CCM on p-ERK 1/2 protein expression in wild-type HS-5 cells after 48 h, as analyzed by immunoblot analysis. GAPDH was used as a housekeeping control. The scale bar in (**B**) is 500 μm. Data in (**D**) are represented as mean ± SEM. Data are representative of at least three (n = 3) separate experiments on distinct passages.**p* < 0.05 as compared to control using a one-way ANOVA, followed by a Tukey’s post-hoc analysis.


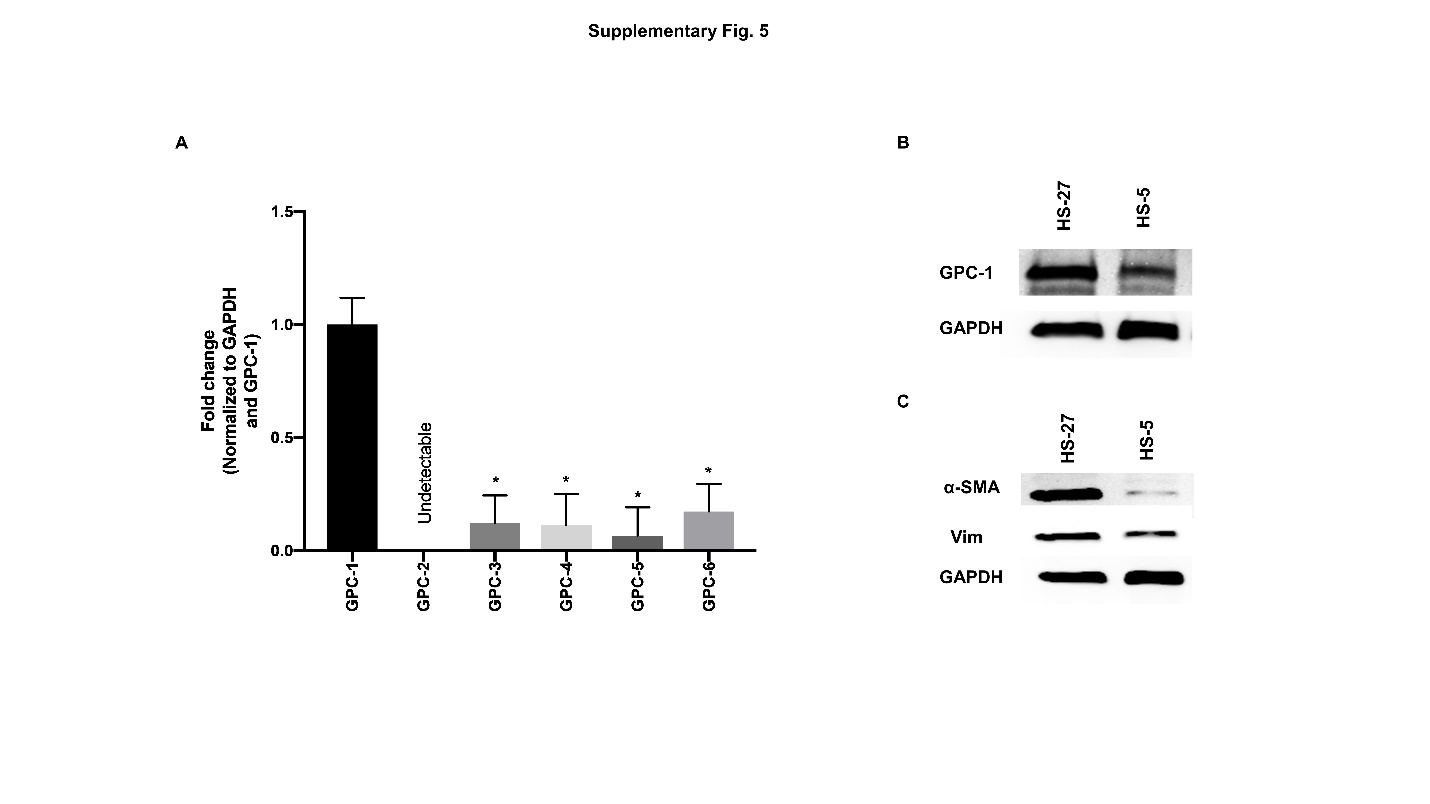


**Supplementary Fig. 5: Expression of GPC isoforms in HS-5 cells, and GPC-1 and CAF markers protein expression in HS-5 and HS-27 cells.** (**A)** Fold change relative to GPC-1 of GPC isoforms in HS-5 cells, as determined by qRT-PCR. GAPDH was used as a housekeeping control. (**B**) GPC-1 and (**C**) CAF markers, α-smooth muscle actin (α-SMA) and vimentin (Vim), protein expression in HS-27 and HS-5 cells, as determined by immunoblot analysis. Data in (**A**) are represented as mean ± SEM. Data are representative of at least three (n = 3) separate experiments. **p*<0.05 as compared to control using one-way ANOVA, followed by a Tukey’s post-hoc analysis.

**
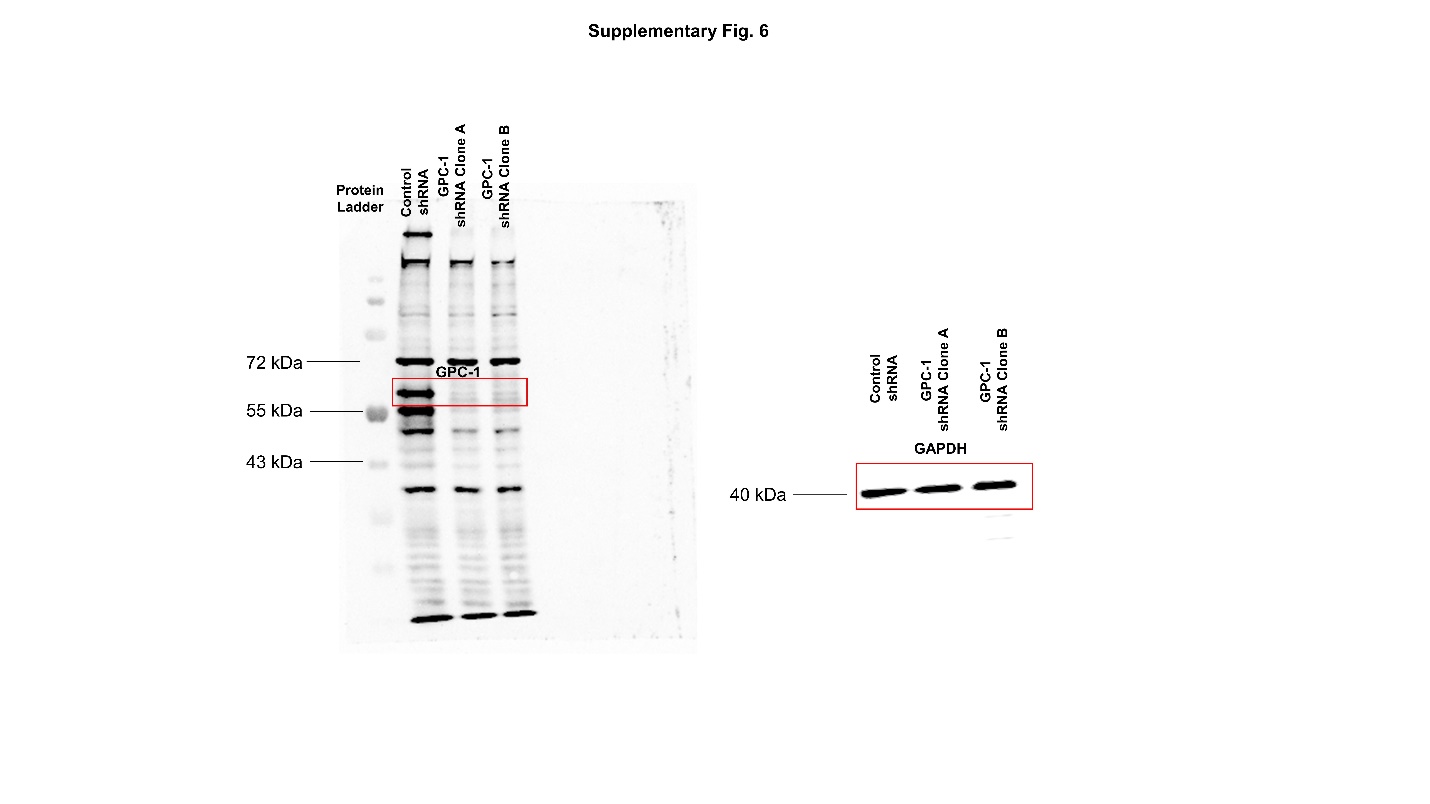
**

**Supplementary Fig. 6:** **Full immunoblot images of Fig. 1B.**

**
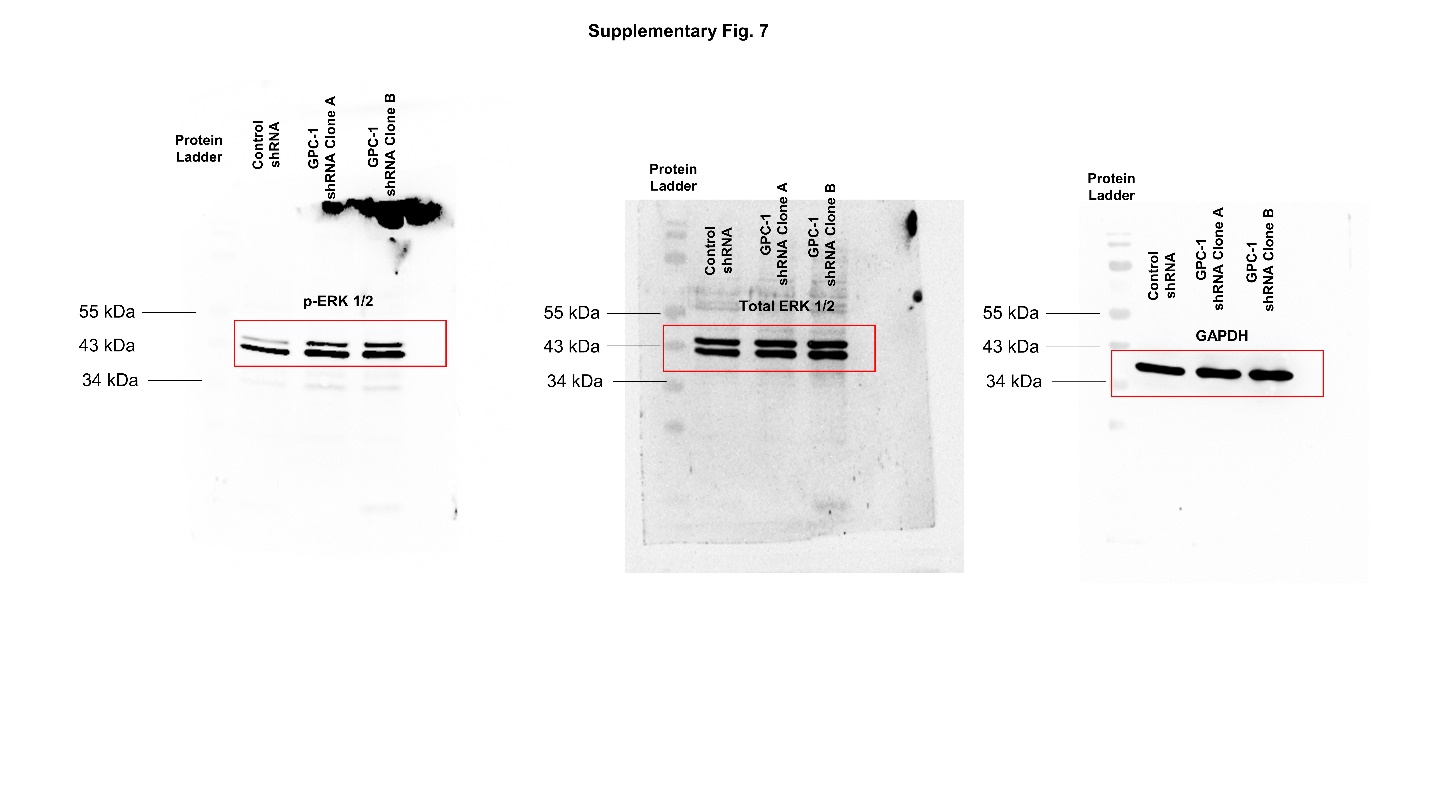
**

**Supplementary Fig. 7: Full immunoblot images of Fig. 3B.**

**
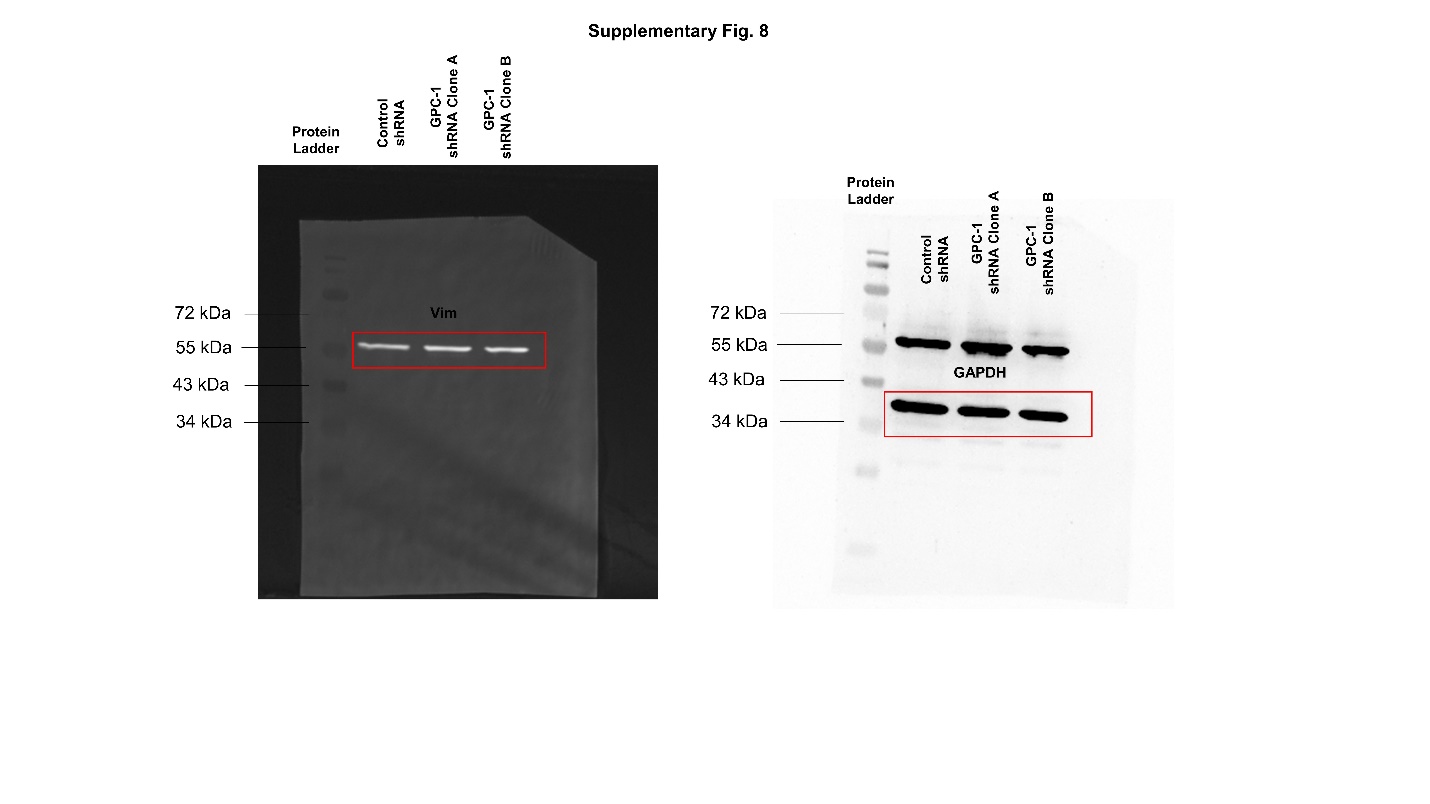
**

**Supplementary Fig. 8: Full immunoblot images of Fig. 3D.**

**
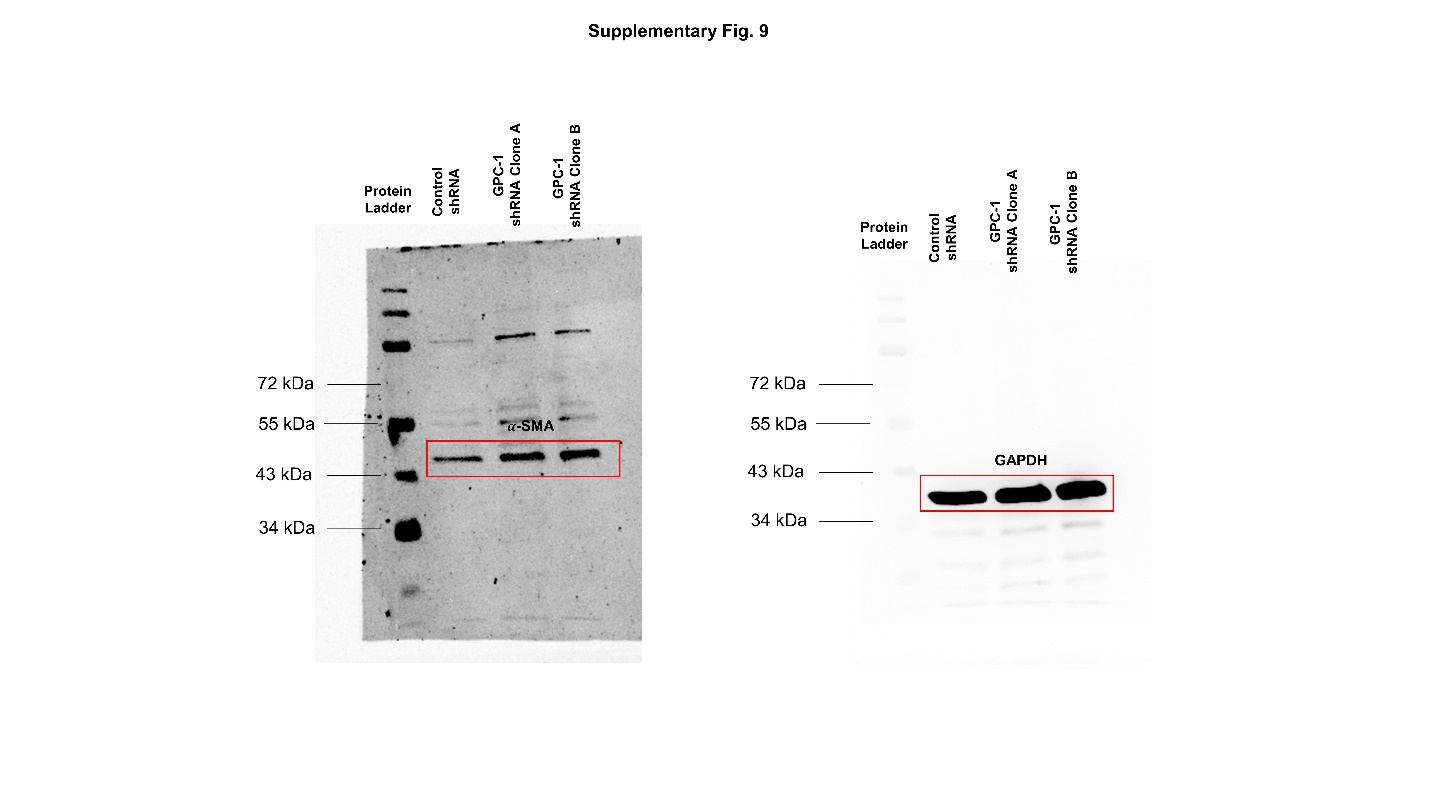
**

**Supplementary Fig. 9: Full immunoblot images of Fig. 3F.**

**
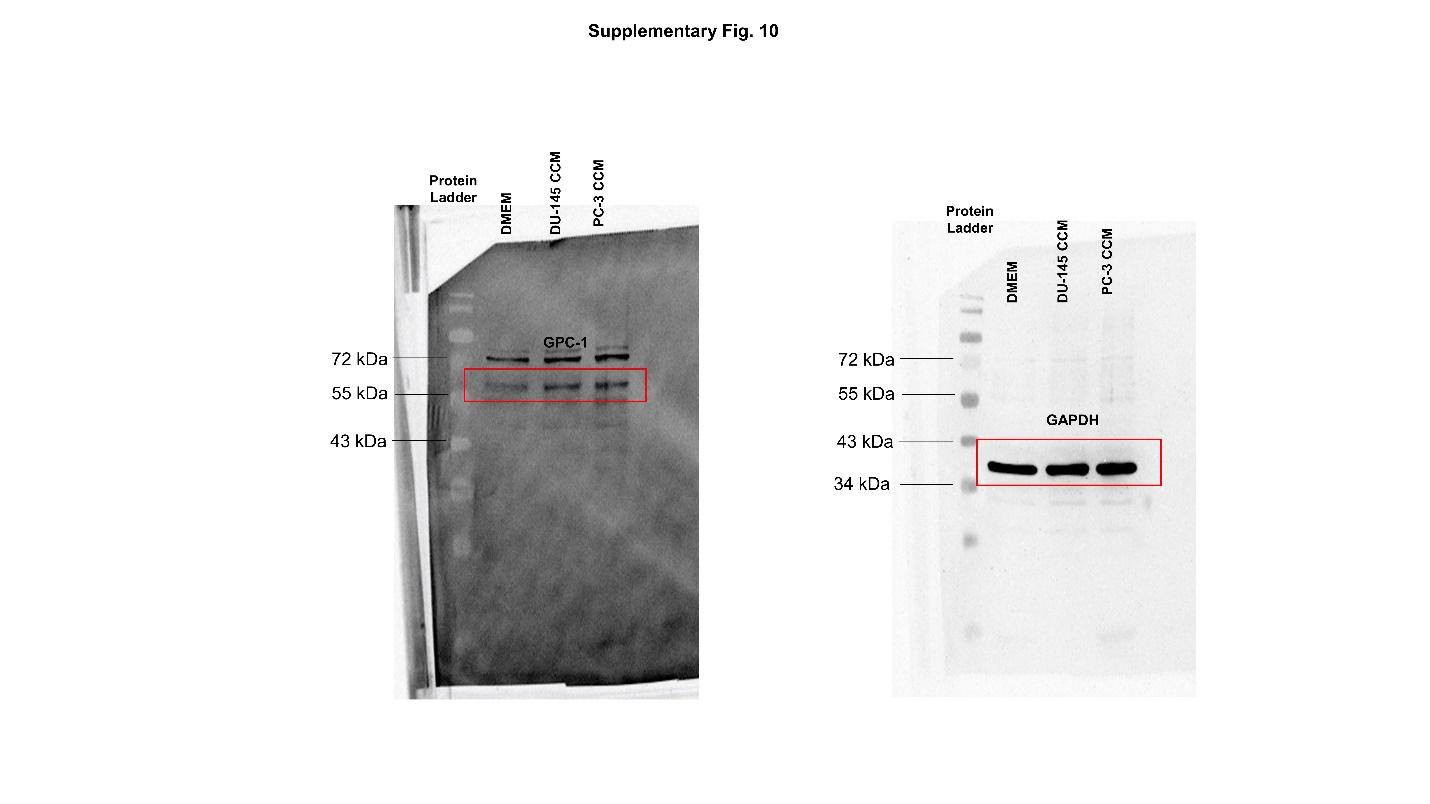
**

**Supplementary Fig. 10: Full immunoblot images of Supplementary Fig. 4A.**

**
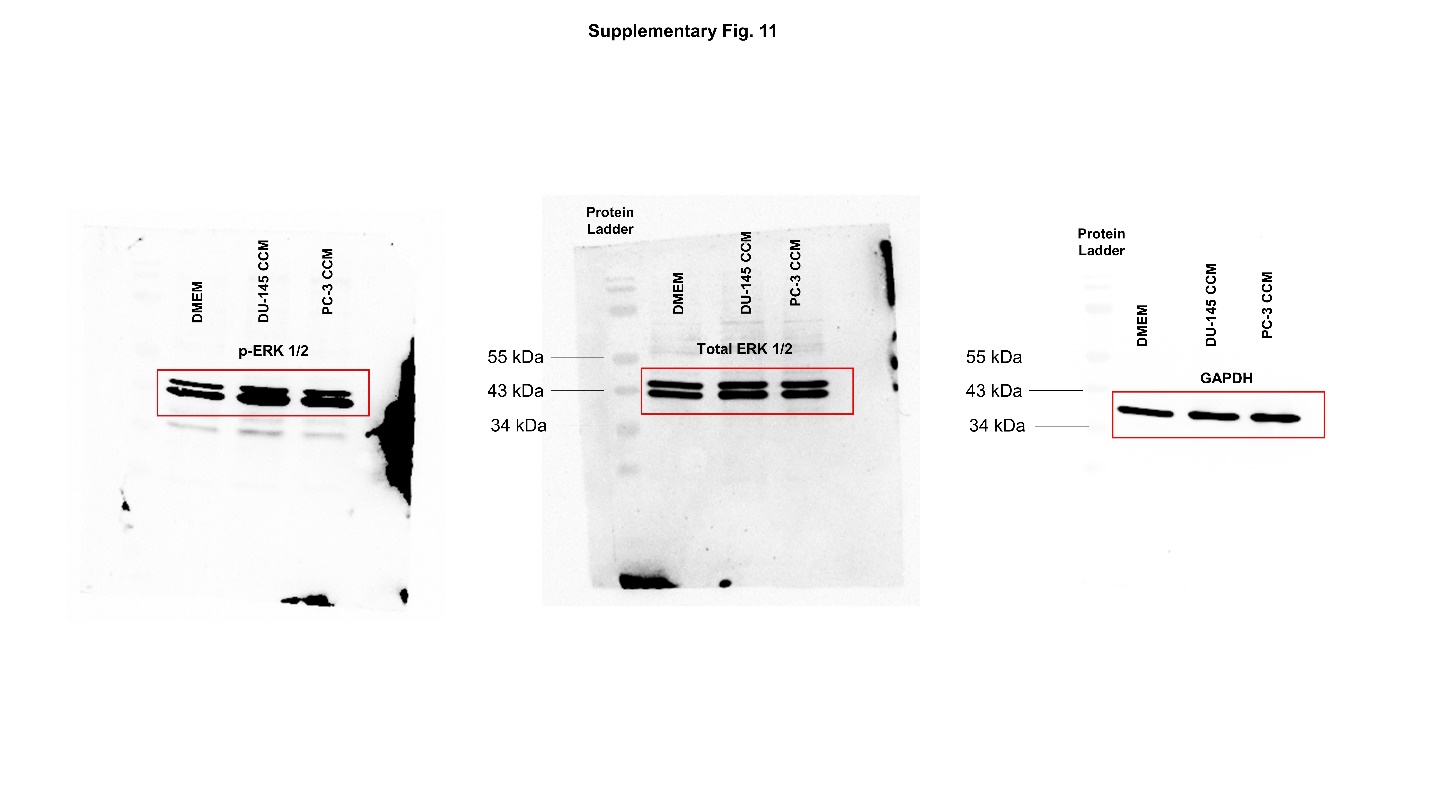
**

**Supplementary Fig. 11: Full immunoblot images of Supplementary Fig. 4E.**

**
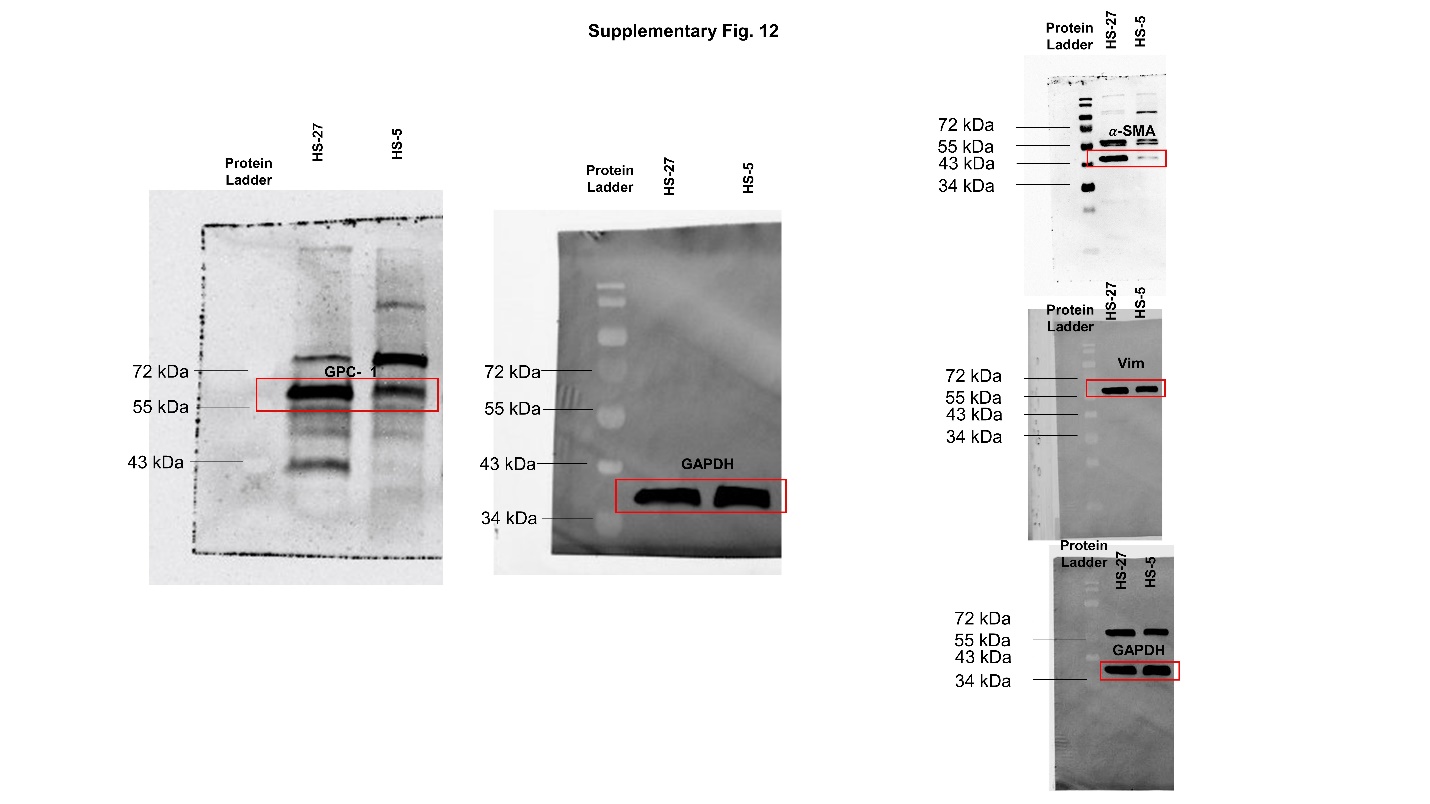
**

**Supplementary Fig. 12: Full immunoblot images of Supplementary Fig. 5, B and C.**
